# Survival Among Veterans Receiving Steroids for Immune-Related Adverse Events After Immune Checkpoint Inhibitor Therapy

**DOI:** 10.1001/jamanetworkopen.2023.40695

**Published:** 2023-10-31

**Authors:** Inga Van Buren, Cecelia Madison, Aimee Kohn, Elizabeth Berry, Rajan P. Kulkarni, Reid F. Thompson

**Affiliations:** 1 Graduate Medical Education, St Joseph’s Medical Center, Stockton, California; 2 Research and Development, VA Portland Healthcare System, Portland, Oregon; 3 Division of Hematology and Medical Oncology, Oregon Health & Science University, Portland; 4 Department of Dermatology, Oregon Health & Science University, Portland; 5 Operative Care Division, VA Portland Healthcare System, Portland, Oregon; 6 Department of Radiation Medicine, Oregon Health & Science University, Portland; 7 Division of Hospital and Specialty Medicine, VA Portland Healthcare System, Portland, Oregon

## Abstract

**Importance:**

Systemic steroids are commonly used to manage immune-related adverse events (irAEs), but it remains unclear whether they may undermine immune checkpoint inhibitor (ICI) therapy outcomes. Few studies have assessed the impact of steroid timing and its association with continuation or cessation of ICI therapy.

**Objective:**

To characterize how systemic steroids and steroid timing for irAEs are associated with survival in patients receiving ICI therapy.

**Design, Setting, and Participants:**

This multicenter retrospective cohort study encompassed veterans receiving ICI for cancer between January 1, 2010, and December 31, 2021. Data analysis was conducted September 8, 2023.

**Exposures:**

Identifiable primary diagnosis of cancer. Patients were categorized into 3 cohorts: those receiving no steroids, systemic steroids for irAEs, and steroids for non–irAE-associated reasons. All eligible patients received 1 or more doses of an ICI (atezolizumab, avelumab, cemiplimab, durvalumab, ipilimumab, nivolumab, or pembrolizumab). Eligible patients in the steroid group received at least 1 dose (intravenous, intramuscular, or oral) of dexamethasone, hydrocortisone, methylprednisolone, prednisone, or prednisolone. Steroid use at baseline for palliation or infusion prophylaxis or delivered as a single dose was deemed to be non–irAE associated. All other patterns of steroid use were assumed to be for irAEs.

**Main Outcomes and Measures:**

The primary outcome was overall survival, with a 5-year follow-up after ICI initiation. Kaplan-Meier survival analyses were performed with pairwise log-rank tests to determine significance. Risk was modeled with Cox proportional hazard regression.

**Results:**

The cohort consisted of 20 163 veterans receiving ICI therapy including 12 221 patients (mean [SD] age, 69.5 [8.0] years; 11 830 male patients [96.8%]; 9394 White patients [76.9%]) who received systemic steroids during ICI treatment and 7942 patients (mean [SD] age, 70.3 [8.5] years; 7747 male patients [97.5%]; 6085 White patients [76.6%]) who did not. Patients with an irAE diagnosis had significantly improved overall survival (OS) compared with those without (median [IQR] OS, 17.4 [6.6 to 48.5] months vs 10.5 [3.5 to 36.8] months; adjusted hazard ratio, 0.84; 95% CI, 0.81-0.84; *P* < .001). For patients with irAEs, systemic steroids for irAEs were associated with significantly improved survival compared with those who received steroids for non–irAE-related reasons or no steroid treatment (median [IQR] OS, 21.3 [9.3 to 58.2] months vs 13.6 [5.5 to 33.7] months vs 15.8 [4.9 to not reached] months; *P *<.001). However, among those who received steroids for irAEs, early steroid use (<2 months after ICI initiation) was associated with reduced relative survival benefit vs later steroid use, regardless of ICI continuation or cessation following steroid initiation (median [IQR] OS after ICI cessation 4.4 [1.9 to 19.5] months vs 16.0 [8.0 to 42.2] months; median [IQR] OS after ICI continuation, 16.0 [7.1 to not reached] months vs 29.2 [16.5 to 53.5] months; *P* <.001).

**Conclusions and Relevance:**

This study suggests that steroids for irAE management may not abrogate irAE-associated survival benefits. However, early steroid administration within 2 months of ICI initiation is associated with shorter survival despite continuation of ICI therapy.

## Key Points

**Question:**

Are systemic steroids and the timing of their administration for immune-related adverse event (irAE) management associated with survival outcomes in patients receiving immune checkpoint inhibitor (ICI) therapy?

**Findings:**

In this cohort study with 20 163 Veterans Health Administration patients, treatment with systemic steroids for irAEs was associated with significantly improved survival compared with nonsteroid treatment or steroid treatment for non–irAE-related reasons. However, patients with early steroid use (<2 months after ICI initiation) had relatively decreased survival compared with those with later use despite continued ICI therapy.

**Meaning:**

Systemic steroids for irAE management were not associated with worsened survival, but the timing of steroid administration matters, and delayed steroid use (≥2 months after ICI therapy initiation) was associated with greatest survival outcomes regardless of ICI continuation or cessation.

## Teaser

This cohort study analyzes the association of systemic steroids for immune-related adverse events (irAEs) with survival among patients with cancer who have received immune checkpoint inhibitor (ICI) therapy.

## Introduction

Immune checkpoint inhibitors (ICIs) have become a cornerstone of oncologic treatment since the approval of ipilimumab for metastatic melanoma.^1^ By modulating T-cell interactions with tumor cells via cytotoxic T-lymphocyte antigen-4 (CTLA-4), programmed cell death 1 (PD-1), or programmed cell death ligand 1 (PDL-1), ICIs enhance antitumor T-cell activity, with promising clinical outcomes.^1^ Unfortunately, ICI treatment is often associated with immune-related adverse events (irAEs) due to blockade of negative regulatory pathways that limit autoimmunity.^2,3^ These irAEs can affect various organ systems and most commonly involve the skin, gastrointestinal tract, endocrine glands, lungs, or liver.^3^

Systemic corticosteroids are the clinical standard for management of most grade 2 or higher irAEs,^4^ but they have classically played a larger role in alleviating cancer-related symptoms (eg, symptomatic brain metastases and pain) for supportive prophylaxis for cancer-specific treatments (eg, chemotherapy-induced nausea or vomiting, and drug infusion reactions) and for comorbid conditions (eg, chronic obstructive pulmonary disease [COPD] and autoimmune disease).^2,5^

Due to their immunosuppressive properties, there is concern that concurrent steroids may undermine the antitumor efficacy of ICIs. Although multiple retrospective studies^6,7^ have suggested that steroids are not associated with decreased overall survival (OS), others have suggested an increased risk of death and cancer progression.^8,9,10,11^ Further subgroup analyses have demonstrated that steroids for irAE management or other non–cancer-related indications are not associated with decreased survival, whereas palliative steroid use is associated with worse outcomes.^8,12,13,14^ Although the association of steroid timing with ICI initiation is potentially contributory, there remain inconsistencies in current data, with some studies showing adverse outcomes with early use or baseline use and others showing no difference.^5,9,13,15^

Given the conflicting evidence, more research is needed to elucidate the association of steroids with cancer survival outcomes and identify optimal strategies for their use in patients receiving ICI therapy. To investigate how steroid administration and its timing is associated with survival, we conducted a retrospective study of US veterans receiving ICIs using the Veterans Health Administration’s (VA) Corporate Data Warehouse (CDW).

## Methods

### Study Population and Subgroups

**Figure 1.**
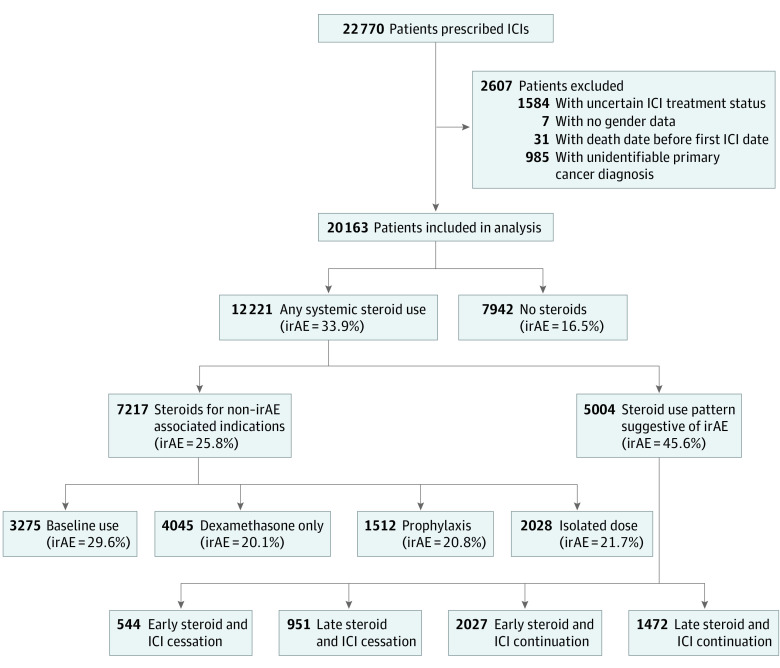
Patient Enrollment Flowchart The figure shows patients prescribed immune checkpoint inhibitors (ICIs) and the allocation of patients who did or did not use steroids. Percentages represent proportion of patients found to have an *International Classification of Diseases, Ninth Revision (ICD-9) *or *International Classification of Diseases, Tenth Revision (ICD-10) *code for an immune-related adverse event (irAE). Patients with uncertain ICI treatment status included those without documented ICI dates, those participating in research studies and potentially receiving placebos, and those who may have only undergone immunohistochemistry staining. The non–irAE steroid subgroups are not mutually exclusive, and some patients in these subgroups nevertheless had an *ICD-9* or *ICD-10* irAE indication.

This cohort study followed the Strengthening the Reporting of Observational Studies in Epidemiology (STROBE) reporting guideline^31^ and was approved by the Portland VA Medical Center institutional review board with a waiver of informed consent because the study used deidentified data in accordance with 45 CFR § 46. We performed a retrospective analysis using the electronic health records available within the VA CDW. The overall process of cohort construction is depicted in Figure 1. Inclusion criteria required patients to receive at least 1 treatment between January 1, 2010, and December 31, 2021, with any of the following ICIs: atezolizumab, avelumab, cemiplimab, durvalumab, ipilimumab, nivolumab, or pembrolizumab. Patients were excluded if they did not have an identifiable primary cancer diagnosis. Those with uncertain ICI treatment status, including patients in research studies potentially receiving placebos, those with only immunohistochemistry staining, and those lacking documented ICI treatment dates, or an otherwise implausible ICI treatment start date (eg, after death), were also excluded. ICI agents were identified using *Current Procedural Terminology* and orderable item codes. Using custom Structured Query Language (SQL) scripts, eligible veterans meeting all inclusion and exclusion criteria were identified across 130 VA Medical Centers.

Our cohort was then divided into a steroid and nonsteroid group. *Current Procedural Terminology* codes were used to identify patients who received systemic steroids following ICI initiation up to 3 months after their last treatment date. Steroids included prednisone, prednisolone, dexamethasone, methylprednisolone, and hydrocortisone. Only steroids that were administered intravenously, intramuscularly, or orally were included. For purposes of a subgroup analysis, we classified each patient on the basis of the most frequent steroid they received, with 2 or more steroids indicating a tie in frequency.

We identified patients with non–irAE-related steroid use, defined as those with baseline-, palliative-, or infusion-related prophylactic use, as well as those who only received a single dose of steroids during the time frame specified. Baseline steroid use referred to systemic steroids within 1 month prior to initiation of ICI therapy with continued use throughout treatment. Patients receiving only dexamethasone were deemed to have palliative steroid use because dexamethasone is less commonly used for management of irAEs.^4,16,17^ Prophylactic steroid use referred to steroid administration 1 day before and up to 3 days after each ICI treatment, which likely represents premedication for infusion related symptoms.^2,18,19^

The remaining patients in the steroid cohort who did not meet the aforementioned criteria were deemed to represent patients who received systemic steroids for irAE treatment. Manual review of 10 randomly selected medical records by a single clinician (I.V.B.) without blinding confirmed that 80% of these patients received systemic steroids for irAE management, whereas 20% received steroids for non–irAE-related reasons (eg, COPD exacerbation). Note that sensitivity analysis allowing up to 20% misattribution of steroid indication demonstrated no effect on our reported results (data not shown).

A group of *International Classification of Diseases, Ninth Revision (ICD-9)* and *International Statistical Classification of Diseases and Related Health Problems, Tenth Revision (ICD-10)* codes (eTable 1 in Supplement 1) deemed to be commonly associated with irAEs was used to orthogonally identify a subgroup of patients with evidence of irAEs in our cohort.^20^ Only diagnoses that first occurred after ICI initiation and up to 3 months after the last treatment date were considered as potential irAEs. To check our inferences, manual medical record review performed by a single clinician (I.V.B.) without blinding confirmed that 8 of 10 randomly selected cases among the patients in the steroids for irAE group having a confirmed irAE *ICD-9 *or *ICD-10* codes received systemic steroids for irAE.

To explore the association of ICI targets with survival outcomes, patients were grouped by anti–PD-1, anti–PD-L1, or anti–CTLA-4 monotherapy. If patients received both an anti–PD-L1 and anti–CTLA-4 agent within an overlapping time frame, they were classified as having received combination therapy. A small subset of patients transitioned between anti–PD-1 and anti–PD-L1 agents and were omitted from this subgroup analysis.

We divided the steroid cohort into 4 additional subgroups to assess timing of steroid administration in relation to ICI continuation status: early or late steroid use with ICI continuation or cessation. Early steroid use was defined as steroid administration less than 2 months after ICI initiation, whereas late steroid use was defined as steroid administration 2 or more months after ICI initiation.^5^ ICI status was defined as continuation or cessation of ICI treatment after steroid initiation (Figure 1).

Custom SQL (SQL Server 2019) and R statistical software version 4.1.2 (R Project for Statistical Computing)^21^ scripts were used to identify all cancer-related *ICD-9* and *ICD-10* codes for our cohort. To determine the primary cancer diagnosis, cancers were first limited to those eligible for ICI treatment and then further limited to those documented 1 month before and 3 months after a patient started ICI treatment. Custom SQL scripts identified the diagnosis made closest to the patient’s ICI start date and the diagnosis documented most frequently during the above time frame. If the same diagnosis was identified through these 2 methods, it was presumed to be the primary cancer diagnosis. Those with a mismatched or unidentifiable diagnosis were excluded (Figure 1). Patients receiving cemiplimab were presumed to have squamous cell carcinoma of the skin because this was its original indication, and this diagnosis was confirmed by manual medical record review in 18 of 18 randomly selected cases as assessed by a single clinician (I.V.B.) without blinding. Finally, *ICD-9* and *ICD-10* codes were used to identify patients with secondary, lymph node, or central nervous system (CNS) metastases, as well as group cancer diagnoses into the categories depicted in the Table and eTable 2 in Supplement 1. Characteristics of the overall nonsteroid and steroid groups are shown in the Table. Characteristics of the steroid subgroups and metastatic data across cancer types are available in eTables 3 and 4 in Supplement 1.

**Table.** Characteristics of the Veteran Cohort

Characteristic	Patients, No. (%) (N = 20 163)		*P* value
	No steroid use (n = 7942)	Any systemic steroid use (n = 12 221)	
Age at first ICI treatment, mean (SD) [range], y	70.3 (8.5) [26-98]	69.5 (8.0) [20-98]	<.001
Sex			
Male	7747 (97.5)	11 830 (96.8)	.002
Female	195 (2.5)	391 (3.2)	NA
Body mass index, mean (SD) [range]^a^	26.7 (5.8) [15-50]	26.9 (5.6) [15-50]	.04
No. of ICI treatments, mean (SD) [range]	8.5 (11.1) [1-137]	11.7 (13.5) [1-140]	<.001
Charlson Comorbidity index score, mean (SD) [range]	12.8 (4.0) [1-31]	12.8 (3.9) [2-30]	.68
Immune-related adverse event code present	1313 (16.5)	4148 (33.9)	<.001
Race			
African American or Black	1213 (15.3)	1973 (16.1)	.10
American Indian or Alaska Native	46 (0.6)	70 (0.6)	.95
Asian	24 (0.3)	36 (0.3)	.92
Declined or unknown	515 (9.4)	669 (5.5)	.003
Native Hawaiian or Pacific Islander	59 (0.8)	79 (0.6)	.42
White	1213 (15.3)	9394 (76.9)	.69
Predominant steroid^b^			
Dexamethasone	NA	6166 (50.5)	NA
Prednisone	NA	3447 (28.2)	NA
Methylprednisolone	NA	1102 (9.0)	NA
Hydrocortisone	NA	813 (6.7)	NA
Prednisolone	NA	3 (0.02)	NA
≥2 steroids	NA	690 (5.6)	NA
ICI target			
Anti–PD-1 monotherapy	6160 (77.6)	8850 (72.4)	<.001
Anti–PD-L1 monotherapy	1039 (13.1)	1685 (13.8)	.15
Anti–CTLA-4 monotherapy	211 (2.6)	240 (2.0)	.001
Anti–CTLA-4 and PD-L1 combination therapy	357 (4.5)	822 (6.7)	<.001
Mixed^c^	175 (2.2)	624 (5.1)	<.001
Smoking status			
Current or former smoker	5114 (64.4)	7703 (63)	<.001
Never smoker	1588 (20)	2077 (17)	<.001
Primary cancer		()	
Bronchus or lung	3565 (44.9)	7427 (60.8)	<.001
Urinary tract	1377 (17.3)	1576 (12.9)	<.001
Melanoma	1025 (12.9)	1324 (10.8)	<.001
Head and neck	603 (7.6)	728 (6.0)	<.001
Liver	725 (9.1)	488 (4.0)	<.001
Gastroesophageal	249 (3.1)	272 (2.2)	<.001
Colorectal	147 (1.9)	131 (1.1)	<.001
Squamous of skin	81 (1.0)	51 (0.4)	<.001
Mesothelioma	40 (0.5)	58 (0.5)	.77
Merkel	44 (0.6)	52 (0.4)	.19
Hodgkin	39 (0.5)	55 (0.5)	.67
Anal	32 (0.4)	29 (0.2)	.04
Breast	15 (0.2)	30 (0.2)	.41
Metastasis			
Any metastases	5655 (71.2)	10 230 (83.7)	<.001
Lymph node metastases	2684 (33.8)	5237 (42.9)	<.001
Centra nervous system metastases	933 (11.7)	2808 (23.0)	<.001

Abbreviations: CTLA-4, cytotoxic T-lymphocyte antigen–4; ICI, immune checkpoint inhibitor; NA, not available; PD-1, programmed cell death 1; PD-L1, programmed cell death ligand 1.

^a^ Body mass index was calculated as weight in kilograms divided by height in meters squared.

^b^ Predominant steroid refers to the most common steroid received by each patient and ≥2 steroids indicates multiple steroids received at equal frequency.

^c^ Mixed ICI target refers to patients receiving both anti–PD-1 and anti–PD-L1 agents (adjusted significance threshold, *P* < .001).

### Outcomes and Covariates

OS was defined as the time (days) survived during a 5-year follow-up period following ICI initiation. Patients lost to follow-up during the study period (3341 patients) were censored at the time of last recorded follow up.

The Charlson Comorbidity Index score at the beginning of ICI treatment was calculated against each patient’s complete medical record using custom R and SQL scripts adapted from published methods.^22,23,24^ Diagnoses pertaining to the index categories and occurring before the start of ICI therapy were extracted from the database and scored according to the Charlson Comorbidity Index algorithm (eTable 5 in Supplement 1). Smoking status at the time of ICI treatment initiation was assessed using custom R and SQL scripts adapted from published methods.^25,26^

### Statistical Analysis

Demographic characteristics between nonsteroid and steroid groups were compared using 2-sided sample *t* tests, *z* tests, and χ^2^ tests in R.^21^ Kaplan-Meier (KM) survival analyses were performed using the survival (versions 3.5-5)^27,28^ and survminer (version 0.4.9)^29^ packages for R^21^ and custom R and SQL scripts. For KM analyses with 2 or more groups, pairwise log-rank tests were performed to determine significance. Significance thresholds for multiple tests were adjusted via Bonferroni correction where relevant.

Using KM analyses, we explored the association of irAEs, general steroid use, and steroid use for irAE management with OS. These analyses were repeated after stratifying by cancer type and presence of secondary metastases. We further explored the association of irAE type, predominant steroid type, and ICI targets with clinical outcomes. Finally, we conducted a subgroup analysis of steroid timing and ICI continuation status.

Risk was modeled with Cox proportional hazard regression (without interaction terms) in R using the survival package (version 3.5-5).^27,28^ The ggplot2 (version 3.3.6)^30^ package was used for generating graphs. Data were censored according to the last follow-up date or date of death, whichever occurred first. Data analysis was conducted September 8, 2023.

## Results

### Patient Characteristics

We identified 20 163 patients in the VA CDW database who received ICIs and met our inclusion and exclusion criteria (Figure 1), with anti–PD-1 monotherapy being the most common ICI in our cohort. A total of 12 221 patients (60.6%; mean [SD] age, 69.5 [8.0] years; 11 830 male patients [96.8%]; 9394 White patients [76.9%]) received systemic steroids during ICI treatment, while 7942 patients (39.4%; mean [SD] age, 70.3 [8.5] years; 7747 male patients [97.5%]; 6085 White patients [76.6%]) did not. For patients who remained alive throughout the study, the mean (SD) follow-up time was 601 (461) days. Patient demographics were reflective of the overall VA population. Both cohorts exhibited a mean Charlson Comorbidity Index score of 12.8 (SD for the nonsteroid group, 4.0; SD for the steroid group, 3.9) which is associated with a 0% 10-year survival rate. Several other demographic characteristics (eg, age at ICI initiation, sex, number of ICI treatments, and smoking history) were found to be clinically similar but statistically significantly different between groups (Table).

Lung cancer accounted for 54.5% (10 992 patients) of the primary cancers in the cohort, followed by urinary tract cancers (2953 patients [14.7%]) and melanoma (2349 patients [11.7%]). In our cohort, ICIs were used in both the adjuvant and metastatic settings, with the steroid group having an overall higher rate of metastasis (10 230 patients [83.7%]) than the nonsteroid group (5655 patients [71.2%]) (Table).

From the entire cohort, 27.1% of patients (5461 patients) had new diagnoses suggestive of irAEs (eg, dermatitis, colitis, pneumonitis, or endocrinopathies) documented after initiation of ICI treatment. Among these patients, the majority received steroids (4148 patients [76.0%]). Gastrointestinal toxic effects were most common in our cohort (1865 patients [34.2%]), followed by endocrine (1518 patients [27.8%]) and dermatologic toxic effects (1001 patients [18.3%]) (eTable 6 in Supplement 1).

### Association of irAEs With Overall Survival

**Figure 2.**
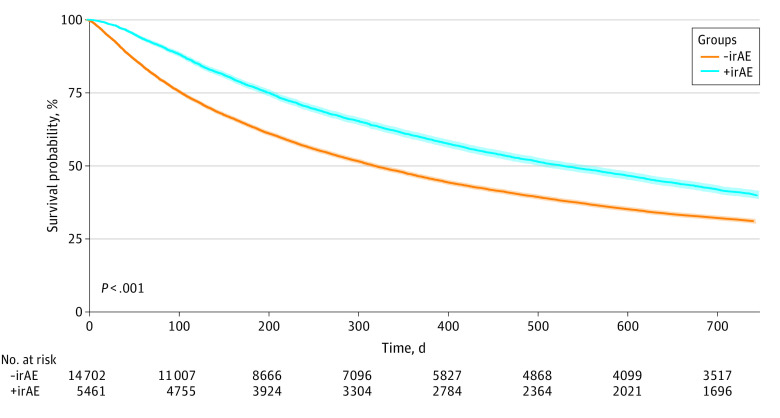
Kaplan-Meier Curve Demonstrating Overall Survival in Patients With and Without Immune-Related Adverse Event (irAE)–Related Indications Survival probability is shown for patients with (+irAE) and without (-irAE) *International Classification of Diseases, Ninth Revision *or *International Classification of Diseases, Tenth Revision *codes for irAEs. The median (IQR) OS was 10.5 (3.5-36.8) months for those without irAE codes and 17.4 (6.6-48.5) months for those with irAE codes (adjusted hazard ratio, 0.84; 95% CI, 0.81-0.84; *P* < .001).

First, we investigated whether the presence of irAEs, identified by *ICD-9 *and *ICD-10* codes, was associated with OS. We found that patients with irAE *ICD-9 *and *ICD-10* codes had significantly increased survival compared with those without irAE-associated *ICD-9 *and *ICD-10* codes (median [IQR] OS, 17.4 [6.6 to 48.5] months vs 10.5 [3.5 to 36.8] months; adjusted HR [aHR], 0.84; 95% CI, 0.81-0.84; *P* < .001) (Figure 2). This difference between patients with irAEs and without irAEs remained significant, independent of cancer type or presence of metastases (eFigures 1-3 in Supplement 1), with a long-term survival benefit most prominent among patients with irAEs with metastases compared with patients without irAEs with metastases. Among patients with irAEs, those who developed dermatologic-related irAEs had some of the best survival outcomes (median [IQR] OS, 26.4 [11.9 to not reached] months) whereas those with hepatic-related irAEs had some of the worst survival outcomes (median [IQR] OS, 6.1 [2.2 to 17.6 months]; *P *<.001) (eFigure 4 in Supplement 1).

### Association of Steroids for irAE Management With OS

**Figure 3.**
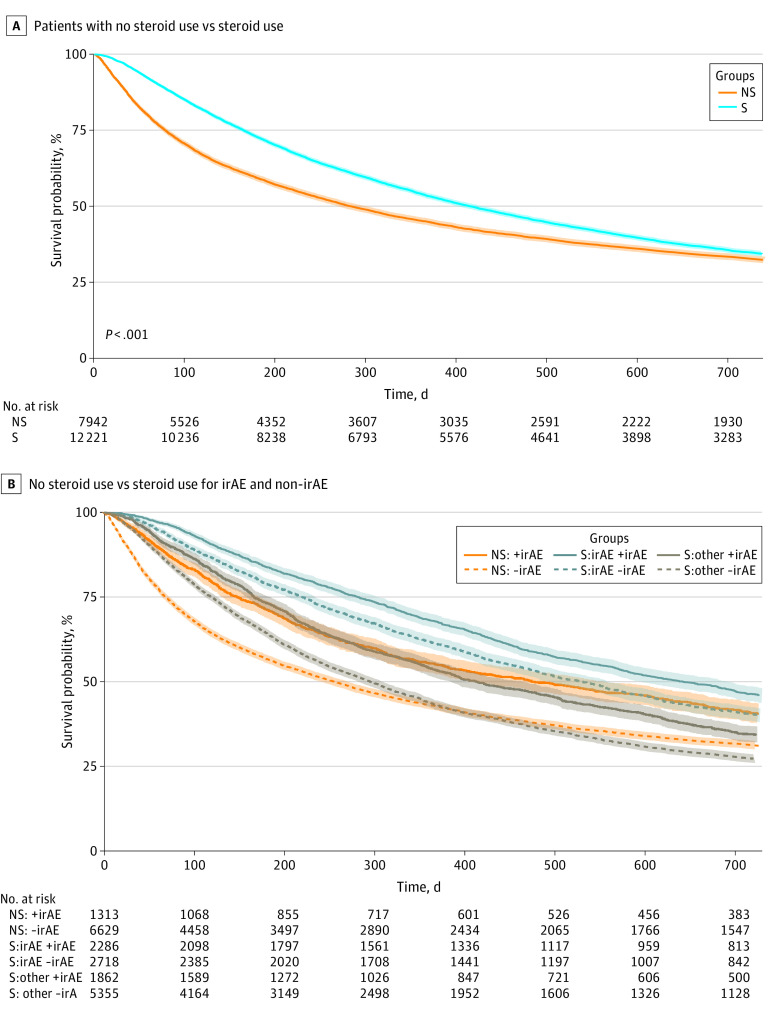
Kaplan-Meier (KM) Curve Showing Association of Steroids With Overall Survival in the Full Veterans Cohort Survival probability is shown for overall steroid (S) and nonsteroid (NS) groups (A) and for the following subgroups: patients in the nonsteroid group who nevertheless had an *International Classification of Diseases, Ninth Revision (ICD-9) *or *International Classification of Diseases, Tenth Revision (ICD-10) *codes for immune-related adverse events (irAEs; NS:+irAE), patients in the nonsteroid group who did not had an *ICD-9 *or *ICD-10* code for irAEs (NS:-irAE), patients receiving steroids for irAEs who had an *ICD-9 *or *ICD-10* code for irAEs (S:irAE+irAE), patients receiving steroids for irAEs who did not have had an *ICD-9 *or *ICD-10* code for irAEs (S:irAE-irAE), patients receiving steroids for non–irAE reasons who nevertheless had an *ICD-9 *or *ICD-10* code for irAEs (S:other+irAE), and patients receiving steroids for non–irAE reasons who did not have an *ICD-9 *or *ICD-10* code for irAEs (S:other-irAE) (B) In Panel A, the adjusted hazard ratio was 0.88 (95% CI, 0.83-0.93; *P* < .001). The adjusted significance threshold was *P* < .003.

To assess how general steroid use was associated with survival, we compared the OS between the nonsteroid and steroid cohorts. The steroid group exhibited a significantly greater early survival benefit over the nonsteroid group, notably during the first year of ICI treatment (median [IQR] OS, 13.7 [5.4-38.8] months vs 9.4 [2.6-50.5] months; aHR, 0.88, 95% CI, 0.83-0.93; *P* < .001) (Figure 3).

In a set of subgroup analyses, we next assessed how irAE development and steroid administration for irAE vs non–irAE indications was associated with OS (Figure 3). Subgroups included the subsets of the nonsteroid group with irAE *ICD-9 *and *ICD-10* codes, nonsteroid group without irAE *ICD-9 *and *ICD-10* codes, steroid group assumed to be receiving steroids for irAE indications who actually had irAE *ICD-9 *and *ICD-10* codes, steroid group assumed to be receiving steroids for irAE indications without irAE *ICD-9 *and *ICD-10* codes, steroid group thought to be receiving steroids for non-irAE indications who nonetheless had irAE-associated *ICD-9 *and *ICD-10* codes, and the steroid group thought to be receiving steroids for a non-irAE indications without irAE *ICD-9 *and *ICD-10* codes.

We observed that subgroups with irAE-associated indications consistently had improved OS compared with subgroups that did not have irAE-associated indications (Figure 3B). The subgroup of patients receiving steroids for irAEs with irAE-associated *ICD-9 *and *ICD-10* codes had the greatest OS (median [IQR] OS, 21.3 [9.3-58.2] months) compared with all other groups. Of note, both subgroups of patients receiving steroids for irAEs (those with *ICD-9 *and *ICD-10* codes and those without) also had significantly greater survival compared with either subgroup of patients receiving steroids for non-irAE indications including those who nonetheless had an irAE-associated *ICD-9 *or *ICD-10* code (median [IQR] OS, 13.6 [5.5-33.7] months) and those without a code (median [IQR] OS, 9.8 [3.9-27.6] months; *P *<.001)(Figure 3B), and this trend persisted in patients with metastases across all cancer types (eFigures 5-9 in Supplement 1). Patients receiving steroids for non-irAE indications cohort had decreased survival compared with nonsteroid patients around 1 year after ICI initiation; however, this was significant for only the patients receiving steroids for non-irAE indications who nonetheless had an irAE *ICD-9 *or *ICD-10* code (Figure 3B). In a stratified analysis, the significantly decreased survival in this group relative to nonsteroid patients was observed only among those with secondary metastases (eFigure 5 in Supplement 1), particularly in patients with metastatic melanoma and urinary tract cancers (eFigures 7 and 8 in Supplement 1).

We also explored the association of steroid type (eFigure 10 in Supplement 1) as well as ICI target (eFigure 11 in Supplement 1) with clinical outcomes in a set of subgroup analyses. Administration predominantly of dexamethasone or 2 or more steroids at equal frequency was associated with the shortest survival (median [IQR] OS, 11.3 [4.9 to 26.4] months and 12.3 [4.3 to 32.3] months, respectively), whereas hydrocortisone was associated with the longest survival (median [IQR] OS, 25.3 [8.9 to not reached] months). Among patients with melanoma or urinary tract cancers, we noted that the association of steroids for irAE management with longer survival had robust ICI target differences (eFigure 11 in Supplement 1). In patients with lung cancer, however, anti–PD-L1 monotherapy without subsequent steroid administration was associated paradoxically with increased survival compared with patients receiving steroids for irAEs (eFigure 11B in Supplement 1). Although the exact explanation for this finding is unclear, we noted that dexamethasone was the only class of steroids associated with relative survival decrement among patients with lung cancer (eFigure 12 in Supplement 1).

### Association of Steroid Timing and ICI Continuation With OS

**Figure 4.**
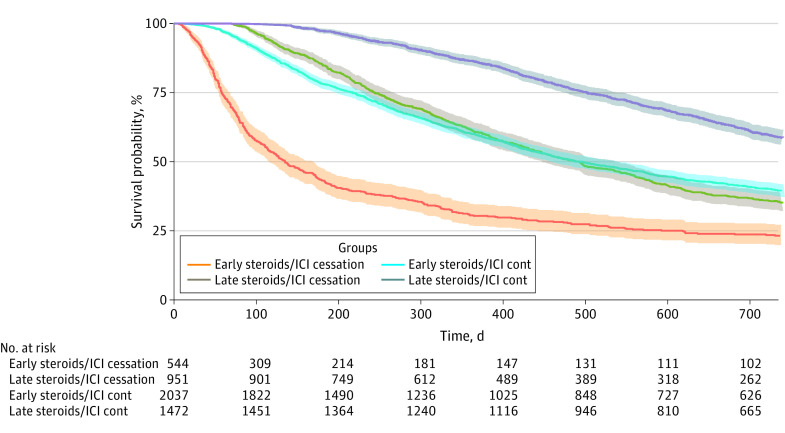
Kaplan-Meier Curve Demonstrating Association of Steroid Timing and Immune Checkpoint Inhibitor (ICI) Continuation Status With Overall Survival (OS) in Patients With an Immune-Related Adverse Event (irAE) Pattern of Steroid Use Early steroid use was defined as steroids administered less than 2 months after ICI initiation, whereas late steroid use was defined as steroids administered 2 or more months after ICI initiation. ICI status was defined as continuation or cessation of ICI treatment after steroid initiation. Solid lines reflect the hazard ratio whereas the shaded areas reflect the 95% CIs. The median (IQR) OS was 4.4 (1.9 to 19.5) months for early steroids and ICI cessation, 16.0 (7.1 to not reached) months for early steroids and ICI continuation, 16.0 (8.0 to 42.2) months for late steroids and ICI cessation, and 29.2 (16.5 to 53.5) months for late steroids and ICI continuation. The adjusted significance threshold was *P* < .008.

Finally, we investigated timing of steroid administration (early vs late use defined as <2 vs ≥2 months) and continuation of ICI treatment by comparing OS in 4 subgroups of the cohort receiving steroids for irAEs: early steroids and ICI continuation, late steroids and ICI continuation, early steroids and ICI cessation, and late steroids and ICI cessation. We found that late steroid use and ICI continuation thereafter were factors associated with a significant survival benefit compared with early steroid use and ICI cessation (median [IQR] OS for late steroid initiation and ICI continuation, 29.2 [16.5 to 53.5] months vs median [IQR] OS for early steroid initiation and ICI cessation, 4.4 [1.9 to 19.5] months) (Figure 4). Even when ICI treatment was continued, patients with early steroid use had significantly decreased OS (median [IQR] OS, 16.0 [17.0 to not reached] months) compared with late steroid use (median [IQR] OS, 29.2 [16.5 to 53.5] months) (Figure 4). An identical trend was also observed for the cohort receiving steroids for non-irAE indications (eFigure 13 in Supplement 1).

## Discussion

To our knowledge, this is the largest cohort study to date investigating how systemic steroid therapy may be associated with OS in patients receiving ICIs, and it is the first to assess steroid timing relative to ICI continuation status following irAE development. Our data support previous studies^5,9,32^ showing improved survival in patients who develop irAEs. Furthermore, we observed improved survival in patients receiving steroids specifically for irAEs. To our knowledge, this analysis is the first to demonstrate an association of the timing of steroid administration in relation to ICI continuation status with OS.

### Systemic Steroids for irAE Management and irAE–Associated Survival Benefits

Our unstratified analysis supports previous studies^2,7,14^ showing that administering steroids for irAEs is not associated with decreased OS compared with nonsteroid groups. Although steroids can modulate T-cell pathways associated with ICI resistance,^8,10^ it is possible that steroids do not abrogate an effective antitumor response once successfully induced by ICIs. Dosage may also play an important role, and prior models have speculated that steroids have immunostimulatory effects at low concentrations but suppressive effects at high concentrations.^33^ Certain doses of glucocorticoid were shown to downregulate PD-L1 pathways in vitro, thereby suppressing T-cell exhaustion and increasing response to ICI.^34^ Interestingly, although low-dose dexamethasone was reported to be a potent inhibitor of PD-L1 pathways, we did not see clinical evidence of this reflected in our lung cancer cohort and acknowledge that there may be many confounding factors at play in this case.

### Timing of Steroid Administration and irAE–Associated Survival Benefits

Despite the survival benefit noted in patients with irAE-associated steroid use, subgroup analyses revealed the nuanced association of steroid timing with survival. For instance, our data align with those of a prior retrospective study^5^ that found that steroid administration less than 2 months after ICI initiation was associated with significantly lower progression-free survival and OS. In mice, early steroid administration was shown to be associated with regrowth of initially responsive tumors.^35^ Among a small cohort of patients with melanoma receiving CTLA-4 blockade, early steroid administration was associated with decreased OS.^35^ The absence of steroid administration early in treatment may allow time for a durable antitumor response to develop and persist despite later steroid exposure. Indeed, steroids were previously shown to inhibit naïve T cells without impairing the antitumor activity of activated T cells.^36^ Alternatively or additionally, the poorer survival associated with early steroid use and ICI cessation may reflect more severe and life-limiting irAEs; however, we were unable to investigate this hypothesis in the current study.

### Non–irAE-Related Steroid Use

Prior studies^13,37^ have reported that steroids for non–cancer-related indications (eg, COPD exacerbations) do not decrease OS; however, steroids for cancer-related palliation can be an independent factor associated with decreased OS and progression-free survival, likely due to their association with advanced disease.^14^ In our subgroup analyses stratified by presence of metastatic disease, we found that patients receiving steroids for non-irAE indications with certain metastatic cancers (eg, metastatic melanoma) had significantly worse survival compared with their nonsteroid counterparts. One possible explanation is that patients with those cancers may have a greater frequency of CNS metastasis and palliative steroid use. Indeed, in our cohort, patients with melanoma had the highest rate of CNS metastasis compared with all other cancers (eTable 4 in Supplement 1). Because patients receiving steroids for non-irAE indications without metastases did not have significantly worse survival compared with their nonsteroid counterparts, this suggests prior associations of palliative steroid use with poor survival may extend to the ICI context.^14^

### ICI Continuation vs Cessation

Existing literature is mixed regarding potential benefits of ICI continuation following irAE development. Some studies report survival benefit with retreatment only in initial nonresponders prior to irAE development,^38,39^ whereas others report no survival difference between retreatment and discontinuation cohorts regardless of initial response.^40^ We were unable to assess ICI response or whether poorer outcomes in the ICI discontinuation group may be associated with irAE-related morbidity or disease progression.

### Limitations

Our study has several limitations. Due to its retrospective design, we cannot distinguish between association vs causation. Administrative databases also inherently contain missing or inaccurate data, potentially reducing the accuracy of our cohort definitions. For instance, our cohort of patients receiving steroids for non-irAE indications may have inadvertently captured some patients with irAE-associated steroid use (particularly among those with an irAE *ICD-9 *or *ICD-10* code). Our identification of irAEs also likely represents an undercount of events because the lack of irAE-related *ICD-9 *and *ICD-10* codes does not preclude presence of actual irAEs. Given the large scale of our study, manual medical record review was not feasible to assess or confirm cohort definitions. We also confined attention here to systemic steroids and, therefore, did not assess other immunosuppressants used in irAE management (eg, infliximab or methotrexate); the exclusion of topical steroids may mean that we enriched for more severe irAEs.

There is potentially limited generalizability to our study because we focused on a cohort of veterans, a primarily male population with a high rate of multiple comorbidities. We also did not account for other latent covariates that could be associated with survival in our cohort (eg, cause of death, number of irAEs developed, irAE grade, tumor molecular subtypes, tumor mutational burden, dose and/or duration of steroid use, changes in body composition with treatment, or immunosuppression or pre-existing autoimmunity). In addition, we did not assess either how ICI patterns of use changed over time or facility-level and geographical variations in practice patterns or event rates. The covariates reported in our Cox model also demonstrated changing proportional hazards over time; thus, all HRs reported reflect averages.

## Conclusions

To our knowledge, this is the largest study to date investigating how irAEs and systemic steroid therapy are associated with OS in patients receiving ICI therapy. This study found that (1) improved survival was associated with patients who developed irAEs compared with those that did not, (2) steroids for management of irAEs were not associated with worse OS, and (3) steroid administration within 2 months of ICI initiation can reduce irAE-associated survival benefits, even with ICI continuation. Future studies are warranted to confirm and expand upon these findings in independent cohorts.

## Notes

Supplement 1. **eTable 1.** *ICD* Codes Commonly Associated With Immune Related Adverse Effects (irAEs) **eTable 2.** Cancer *ICD* Codes **eTable 3.** Characteristics of the Veteran Cohort in Steroid Subgroups **eTable 4.** Primary Cancer Diagnoses and Metastasis Data of the Entire VA Cohort **eTable 5.** Charlson Score Calculation **eTable 6.** irAE Types in Patients With irAE–Related *ICD* Codes (+irAE ICD) **eFigure 1.** Secondary Metastases and irAE Diagnosis on Survival **eFigure 2.** irAE Diagnosis and Survival Across Cancer Types **eFigure 3.** Secondary Metastases and irAE Diagnosis on Survival Across Cancer Types **eFigure 4.** irAE Type and Survival **eFigure 5.** Steroid Use and Presence of Metastases on Survival **eFigure 6.** Steroid Use and Presence of Metastases on Survival in Lung Cancer Patients **eFigure 7. **Steroid Use and Presence of Metastases on Survival in Melanoma Patients **eFigure 8.** Steroid Use and Presence of Metastases on Survival in Urinary Cancer Patients **eFigure 9.** Steroid Use and Presence of Metastases on Survival in Patients With All Other Cancers **eFigure 10.** Predominant Steroid Type and Survival **eFigure 11.** ICI Target and Steroid Use on Survival Across Cancer Types **eFigure 12.** Steroid Type and Survival in Lung Cancer Patients Receiving Anti–PD-L1 Monotherapy **eFigure 13.** Steroid Timing and ICI Continuation Status on Survival in S:Other Patients

Supplement 2.
Data Sharing Statement
