# Supplementary material for: Survival Among Veterans Receiving Steroids for Immune-Related Adverse Events After Immune Checkpoint Inhibitor Therapy
Source: JAMA Netw Open. 2023 Oct 31;6(10):e2340695. doi: 10.1001/jamanetworkopen.2023.40695 (PMC10618850; doi:10.1001/jamanetworkopen.2023.40695)
Supplement: Supplement 1. — eTable 1. ICD Codes Commonly Associated With Immune Related Adverse Effects (irAEs) eTable 2. Cancer ICD Codes eTable 3. Characteristics of the Veteran Cohort in Steroid Subgroups eTable 4. Primary Cancer Diagnoses and Metastasis Data of the Entire VA Cohort eTable 5. Charlson Score Calculation eTable 6. irAE Types in Patients With irAE–Related ICD Codes (+irAE ICD) eFigure 1. Secondary Metastases and irAE Diagnosis on Survival eFigure 2. irAE Diagnosis and Survival Across Cancer Types eFigure 3. Secondary Metastases and irAE Diagnosis on Survival Across Cancer Types eFigure 4. irAE Type and Survival eFigure 5. Steroid Use and Presence of Metastases on Survival eFigure 6. Steroid Use and Presence of Metastases on Survival in Lung Cancer Patients eFigure 7. Steroid Use and Presence of Metastases on Survival in Melanoma Patients eFigure 8. Steroid Use and Presence of Metastases on Survival in Urinary Cancer Patients eFigure 9. Steroid Use and Presence of Metastases on Survival in Patients With All Other Cancers eFigure 10. Predominant Steroid Type and Survival eFigure 11. ICI Target and Steroid Use on Survival Across Cancer Types eFigure 12. Steroid Type and Survival in Lung Cancer Patients Receiving Anti–PD-L1 Monotherapy eFigure 13. Steroid Timing and ICI Continuation Status on Survival in S:Other Patients [file jamanetwopen-e2340695-s001.pdf]

Supplemental Online Content

Van Buren I, Madison C, Kohn A, Berry E, Kulkarni RP, Thompson RF. Survival among veterans receiving steroids for immune-related adverse events after immune checkpoint inhibitor therapy. *JAMA Netw Open*. 2023;6(10):e2340695 doi:10.1001/jamanetworkopen.2023.40695

eTable 1. *ICD* Codes Commonly Associated With Immune Related Adverse Effects (irAEs)

***e*Table 2.** Cancer *ICD* Codes

eTable 3. Characteristics of the Veteran Cohort in Steroid Subgroups

eTable 4. Primary Cancer Diagnoses and Metastasis Data of the Entire VA Cohort

eTable 5. Charlson Score Calculation

eTable 6. irAE Types in Patients With irAE–Related *ICD* Codes (+irAE *ICD*)

eFigure 1. Secondary Metastases and irAE Diagnosis on Survival

eFigure 2. irAE Diagnosis and Survival Across Cancer Types

eFigure 3. Secondary Metastases and irAE Diagnosis on Survival Across Cancer Types

eFigure 4. irAE Type and Survival

eFigure 5. Steroid Use and Presence of Metastases on Survival

eFigure 6. Steroid Use and Presence of Metastases on Survival in Lung Cancer Patients

eFigure 7. Steroid Use and Presence of Metastases on Survival in Melanoma Patients

eFigure 8. Steroid Use and Presence of Metastases on Survival in Urinary Cancer Patients **eFigure 9.** Steroid Use and Presence of Metastases on Survival in Patients With All Other Cancers **eFigure 10.** Predominant Steroid Type and Survival

eFigure 11. ICI Target and Steroid Use on Survival Across Cancer Types

eFigure 12. Steroid Type and Survival in Lung Cancer Patients Receiving Anti–PD-L1 Monotherapy

eFigure 13. Steroid Timing and ICI Continuation Status on Survival in S:Other Patients

This supplemental material has been provided by the authors to give readers additional information about their work.

© 2023 Van Buren I et al. *JAMA Network Open.*

eTable 1. ICD codes commonly associated with immune related adverse effects (irAEs)

Category	ICD10	ICD9	Description
Dermatologic	L13.9	694.9	Bullous dermatitis
Dermatologic	L27	-	Dermatitis due to substance taken internally
Dermatologic	L27.0	693.0	Dermatitis due to substance taken internally
Dermatologic	L27.1	693.0	Dermatitis due to substance taken internally
Dermatologic	L27.8	693.8	Dermatitis due to substance taken internally
Dermatologic	L27.9	693.9	Dermatitis due to substance taken internally
Dermatologic	L29	-	Pruritus
Dermatologic	L29.8	698.8	Pruritis other
Dermatologic	L29.9	698.9	Pruritis NOS
Dermatologic	L30.9	692.9	Dermatitis
Dermatologic	L30.9	-	Drug reaction/eruption
Dermatologic	L51	-	Erythema multiforme
Dermatologic	L51.1	695.13	SJS
Dermatologic	L51.2	695.15	TEN
Dermatologic	L51.3	695.14	SJS/TEN overlap
Dermatologic	L51.8	695.11	Other erythema multiforme
Dermatologic	L51.8	695.12	Other erythema multiforme
Dermatologic	L51.8	695.19	Other erythema multiforme
Dermatologic	L51.9	695.10	Erythema multiforme unspecified
Dermatologic	L80	709.01	Vitiligo
Dermatologic	-	692.9	Dermatitis
Dermatologic	-	995.2	Drug reaction/eruption
Dermatologic	-	502.164	Dermatitis due to chemotherapy
Dermatologic	R21	782.1	Rash NOS
Endocrine	E03.2	244.3	Hypothyroidism due to medicaments and other exogenous substances
Endocrine	E03.8	244.8	Other specified hypothyroidism
Endocrine	E03.9	244.9	Hypothyroidism unspecified
Endocrine	E05.80	242.80	Other thyrotoxicosis
Endocrine	E05.81	242.81	Other thyrotoxicosis
Endocrine	E05.90	242.90	Thyrotoxicosis unspecified
Endocrine	E23.0	253.2	Hypopituitarism
Endocrine	E23.0	253.3	Hypopituitarism
Endocrine	E23.0	628.1	Hypopituitarism
Endocrine	E23.1	253.7	Drug-induced hypopituitarism
Endocrine	E23.6	253.4	Other disorders of pituitary gland
Endocrine	E23.6	253.8	Other disorders of pituitary gland
Endocrine	E23.7	253.9	Disorder of pituitary gland unspecified
Endocrine	E27.1	255.41	Primary adrenocortical insufficiency
Endocrine	E27.3	-	Drug-induced adrenocortical insufficiency
Endocrine	E27.40	255.41	Unspecified adrenocortical insufficiency
Endocrine	E27.49	255.42	Other adrenocortical insufficiency
Endocrine	E27.49	255.5	Other adrenocortical insufficiency
Endocrine	E27.8	255.8	Other specified disorders of adrenal gland
Endocrine	E27.9	255.9	Disorder of adrenal gland unspecified
Gastrointestinal	K29.00	535.00	Acute gastritis

© 2023 Van Buren I et al. *JAMA Network Open.*

Gastrointestinal	K29.01	535.01	Acute gastritis
**Category**	**ICD10**	**ICD9**	**Description**
Gastrointestinal	K29.60	535.20	Other gastritis
Gastrointestinal	K29.61	535.21	Other gastritis
Gastrointestinal	K29.70	535.50	Gastritis unspecified
Gastrointestinal	K29.71	535.51	Gastritis unspecified
Gastrointestinal	K29.80	535.60	Duodenitis
Gastrointestinal	K29.81	535.61	Duodenitis
Gastrointestinal	K51.519	556.5	Left sided colitis with unspecified complications
Gastrointestinal	K52.832	558.9	Lymphocytic colitis
Gastrointestinal	K52.839	558.9	Microscopic colitis unspecified
Gastrointestinal	K52.1	558.2	Toxic gastroenteritis and colitis
Gastrointestinal	K52.3	558.9	Indeterminate colitis
Gastrointestinal	K52.89	558.9	Noninfective gastroenteritis and colitis
Gastrointestinal	K52.9	558.9	Enterocolitis
Gastrointestinal	-	787.91	Diarrhea
Gastrointestinal	-	535.40	Other gastritis
Gastrointestinal	-	535.41	Other gastritis
Gastrointestinal	-	536.8	Gastritis/dyspepsia
Gastrointestinal	R19.7	-	Diarrhea
Hepatic	K71	-	Toxic liver disease
Hepatic	K71.6	573.3	Toxic liver disease
Hepatic	K72	-	Hepatic failure NOS
Hepatic	K72.00	570	Hepatic failure NOS
Hepatic	K72.10	572.8	Hepatic failure NOS
Hepatic	K72.90	572.2	Hepatic failure NOS
Hepatic	K75.2	573.3	Nonspecific reactive hepatitis
Hepatic	K75.4	571.42	Autoimmune hepatitis
Hepatic	K75.9	573.3	Inflammatory liver disease NOS
Neurologic	G70.00	358.00	Myasthenia gravis without (acute) exacerbation
Neurologic	G70.01	358.01	Myasthenia gravis with (acute) exacerbation
Neurologic	I67.83	348.39	Posterior reversible encephalopathy syndrome
Neurologic	G04.81	323.81	Other encephalitis and encephalomyelitis
Neurologic	G04.89	323.82	Other myelitis
Neurologic	G04.89	323.9	Encephalitis and encephalomyelitis unspecified
Neurologic	G04.91	323.9	Myelitis unspecified
Pancreatic	K85.3	-	Drug induced pancreatitis
Pancreatic	K85.8	-	Other acute pancreatitis
Pancreatic	K85.9	577.0	Pancreatitis
Pulmonary	J70.2	-	Acute drug-induced interstitial lung disorders
Pulmonary	J70.3	-	Chronic drug-induced interstitial lung disorders
Pulmonary	J70.4	-	Drug-induced interstitial lung disorder unspecified
Pulmonary	J70.8	508.8	Respiratory conditions due to external agents
Pulmonary	J84.113	516.32	Idiopathic non-specific interstitial pneumonitis
Pulmonary	J84.114	516.33	Acute interstitial pneumonitis
Pulmonary	-	486	Pneumonitis
Renal	N00.2	580.0	acute nephritic syndrome with diffuse membranous glomerulonephritis

© 2023 Van Buren I et al. *JAMA Network Open.*

Renal	N00.7	580.0	acute nephritic syndrome with diffuse crescentic glomerulonephritis
Renal	N01.3	580.4	rapidly progressive nephritic syndrome with diffuse crescentic glomerulonephritis
**Category**	**ICD10**	**ICD9**	**Description**
Renal	N05.2	583.1	Unspecified nephritic syndrome
Renal	N05.5	583.2	Unspecified nephritic syndrome
Renal	N05.8	583.89	Unspecified nephritic syndrome
Renal	N05.9	583.0	Unspecified nephritic syndrome
Renal	N10	590.11	Acute pyelonephritis
Renal	N10	590.10	Acute pyelonephritis
Renal	N28.89	593.89	Unspecified nephritic syndrome
Renal	N28.9	593.9	Disorder of kidney and ureter unspecified
Renal	-	583.4	Unspecified nephritic syndrome
Renal	-	583.9	Unspecified nephritic syndrome
Renal	-	583.81	Nephritis
Optho	H44.111	360.12	Panuveitis right
Optho	H44.112	360.12	Panuveitis left
Optho	H44.113	360.12	Panuveitis bilateral
Optho	H44.119	360.12	Panuveitis unspecified eye

© 2023 Van Buren I et al. *JAMA Network Open.*

eTable 2. Cancer ICD codes

eTable 2a. Bronchus/lung cancer ICD codes

Code	Description
162.2	Malignant neoplasm of main bronchus
162.3	Malignant neoplasm of upper lobe, bronchus or lung
162.4	Malignant neoplasm of middle lobe, bronchus or lung
162.5	Malignant neoplasm of lower lobe, bronchus or lung
162.8	Malignant neoplasm of other parts of bronchus or lung
162.9	Malignant neoplasm of bronchus and lung, unspecified
C34.00	Malignant neoplasm of unspecified main bronchus
C34.01	Malignant neoplasm of right main bronchus
C34.02	Malignant neoplasm of left main bronchus
C34.10	Malignant neoplasm of upper lobe, unspecified bronchus or lung
C34.11	Malignant neoplasm of upper lobe, right bronchus or lung
C34.12	Malignant neoplasm of upper lobe, left bronchus or lung
C34.30	Malignant neoplasm of lower lobe, unspecified bronchus or lung
C34.31	Malignant neoplasm of lower lobe, right bronchus or lung
C34.32	Malignant neoplasm of lower lobe, left bronchus or lung
C34.80	Malignant neoplasm of overlapping sites of unspecified bronchus and lung
C34.81	Malignant neoplasm of overlapping sites of right bronchus and lung
C34.82	Malignant neoplasm of overlapping sites of left bronchus and lung
C34.90	Malignant neoplasm of unspecified part of unspecified bronchus or lung
C34.91	Malignant neoplasm of unspecified part of right bronchus or lung
C34.92	Malignant neoplasm of unspecified part of left bronchus or lung

eTable 2b. Urothelial cancer ICD codes

Code	Description
188.0	Malignant neoplasm of trigone of urinary bladder
188.1	Malignant neoplasm of dome of urinary bladder
188.2	Malignant neoplasm of lateral wall of urinary bladder
188.3	Malignant neoplasm of anterior wall of urinary bladder
188.4	Malignant neoplasm of posterior wall of urinary bladder
188.5	Malignant neoplasm of bladder neck
188.6	Malignant neoplasm of ureteric orifice
188.7	Malignant neoplasm of urachus
188.8	Malignant neoplasm of other specified sites of bladder
188.9	Malignant neoplasm of bladder, part unspecified
**Code**	**Description**
189.0	Malignant neoplasm of kidney, except pelvis
189.1	Malignant neoplasm of renal pelvis
189.2	Malignant neoplasm of ureter
189.3	Malignant neoplasm of urethra
189.4	Malignant neoplasm of paraurethral glands
189.8	Malignant neoplasm of other specified sites of urinary organs
189.9	Malignant neoplasm of urinary organ, site unspecified
C64.1	Malignant neoplasm of right kidney, except renal pelvis
C64.2	Malignant neoplasm of left kidney, except renal pelvis
C64.9	Malignant neoplasm of unsp kidney, except renal pelvis
C65.1	Malignant neoplasm of right renal pelvis
C65.2	Malignant neoplasm of left renal pelvis
C65.9	Malignant neoplasm of unspecified renal pelvis
C66.1	Malignant neoplasm of right ureter
C66.2	Malignant neoplasm of left ureter
C66.9	Malignant neoplasm of unspecified ureter
C67.0	Malignant neoplasm of trigone of bladder
C67.1	Malignant neoplasm of dome of bladder

© 2023 Van Buren I et al. *JAMA Network Open.*

C67.2	Malignant neoplasm of lateral wall of bladder
C67.3	Malignant neoplasm of anterior wall of bladder
C67.4	Malignant neoplasm of posterior wall of bladder
C67.5	Malignant neoplasm of bladder neck
C67.6	Malignant neoplasm of ureteric orifice
C67.7	Malignant neoplasm of urachus
C67.8	Malignant neoplasm of overlapping sites of bladder
C67.9	Malignant neoplasm of bladder, unspecified
C68.0	Malignant neoplasm of urethra
C68.1	Malignant neoplasm of paraurethral glands
C68.8	Malignant neoplasm of overlapping sites of urinary organs
C68.9	Malignant neoplasm of urinary organ, unspecified

eTable 2c. Melanoma ICD codes

Code	Description
172	Malignant melanoma of skin of lip
172.1	Malignant melanoma of skin of eyelid including canthus
172.2	Malignant melanoma of skin of ear and external auditory canal
172.3	Malignant melanoma of skin of other and unspecified parts of face
172.4	Malignant melanoma of skin of scalp and neck
172.5	Malignant melanoma of skin of trunk except scrotum
172.6	Malignant melanoma of skin of upper limb including shoulder
172.7	Malignant melanoma of skin of lower limb including hip
172.8	Malignant melanoma of other specified sites of skin
172.9	Melanoma of skin site unspecified
C43.0	Malignant melanoma of lip
C43.1	Malignant melanoma of unspecified eyelid, including canthus
C43.2	Malignant melanoma of unspecified ear and external auricular canal
C43.3	Malignant melanoma of unspecified part of face
C43.31	Malignant melanoma of nose
C43.39	Malignant melanoma of other parts of face
C43.4	Malignant melanoma of scalp and neck
C43.59	Malignant melanoma of other part of trunk
C43.6	Malignant melanoma of unspecified upper limb, including shoulder

eTable 2d. Head and neck cancer ICD codes

Code	Description
140.0	Malignant neoplasm of upper lip, vermilion border
140.1	Malignant neoplasm of lower lip, vermilion border
140.3	Malignant neoplasm of upper lip, inner aspect
**Code**	**Description**
140.4	Malignant neoplasm of lower lip, inner aspect
140.5	Malignant neoplasm of lip, unspecified, inner aspect
140.6	Malignant neoplasm of commissure of lip
140.8	Malignant neoplasm of other sites of lip
140.9	Malignant neoplasm of lip, unspecified, vermilion border
141.0	Malignant neoplasm of base of tongue
141.1	Malignant neoplasm of dorsal surface of tongue
141.2	Malignant neoplasm of tip and lateral border of tongue
141.3	Malignant neoplasm of ventral surface of tongue
141.4	Malignant neoplasm of anterior two-thirds of tongue, part unspecified
141.5	Malignant neoplasm of junctional zone of tongue
141.6	Malignant neoplasm of lingual tonsil
141.8	Malignant neoplasm of other sites of tongue
141.9	Malignant neoplasm of tongue, unspecified
142.0	Malignant neoplasm of parotid gland
142.1	Malignant neoplasm of submandibular gland
142.2	Malignant neoplasm of sublingual gland

© 2023 Van Buren I et al. *JAMA Network Open.*

142.8	Malignant neoplasm of other major salivary glands
142.9	Malignant neoplasm of salivary gland, unspecified
143.0	Malignant neoplasm of upper gum
143.1	Malignant neoplasm of lower gum
143.8	Malignant neoplasm of other sites of gum
143.9	Malignant neoplasm of gum, unspecified
144.0	Malignant neoplasm of anterior portion of floor of mouth
144.1	Malignant neoplasm of lateral portion of floor of mouth
144.8	Malignant neoplasm of other sites of floor of mouth
144.9	Malignant neoplasm of floor of mouth, part unspecified
145.0	Malignant neoplasm of cheek mucosa
145.1	Malignant neoplasm of vestibule of mouth
145.2	Malignant neoplasm of hard palate
145.3	Malignant neoplasm of soft palate
145.4	Malignant neoplasm of uvula
145.5	Malignant neoplasm of palate, unspecified
145.6	Malignant neoplasm of retromolar area
145.8	Malignant neoplasm of other specified parts of mouth
145.9	Malignant neoplasm of mouth, unspecified
146.0	Malignant neoplasm of tonsil
146.1	Malignant neoplasm of tonsillar fossa
146.2	Malignant neoplasm of tonsillar pillars (anterior) (posterior)
146.3	Malignant neoplasm of vallecula epiglottica
146.4	Malignant neoplasm of anterior aspect of epiglottis
146.5	Malignant neoplasm of junctional region of oropharynx
146.6	Malignant neoplasm of lateral wall of oropharynx
146.7	Malignant neoplasm of posterior wall of oropharynx
146.8	Malignant neoplasm of other specified sites of oropharynx
146.9	Malignant neoplasm of oropharynx, unspecified site
147.0	Malignant neoplasm of superior wall of nasopharynx
147.1	Malignant neoplasm of posterior wall of nasopharynx
147.2	Malignant neoplasm of lateral wall of nasopharynx
147.3	Malignant neoplasm of anterior wall of nasopharynx
147.8	Malignant neoplasm of other specified sites of nasopharynx
147.9	Malignant neoplasm of nasopharynx, unspecified site
148.1	Malignant neoplasm of pyriform sinus
148.2	Malignant neoplasm of aryepiglottic fold, hypopharyngeal aspect
148.3	Malignant neoplasm of posterior hypopharyngeal wall
148.8	Malignant neoplasm of other specified sites of hypopharynx
148.9	Malignant neoplasm of hypopharynx, unspecified site
149.0	Malignant neoplasm of pharynx, unspecified
**Code**	**Description**
149.1	Malignant neoplasm of waldeyer's ring
149.8	Malignant neoplasm of other sites within the lip and oral cavity
149.9	Malignant neoplasm of ill-defined sites within the lip and oral cavity
161.8	Malignant neoplasm of other specified sites of larynx
161.9	Malignant neoplasm of larynx, unspecified
C00.0	Malignant neoplasm of external upper lip
C00.1	Malignant neoplasm of external lower lip
C00.2	Malignant neoplasm of external lip, unspecified
C00.3	Malignant neoplasm of upper lip, inner aspect
C00.4	Malignant neoplasm of lower lip, inner aspect
C00.5	Malignant neoplasm of lip, unspecified, inner aspect
C00.6	Malignant neoplasm of commissure of lip, unspecified
C00.8	Malignant neoplasm of overlapping sites of lip
C00.9	Malignant neoplasm of lip, unspecified
C01.	Malignant neoplasm of base of tongue
C02.0	Malignant neoplasm of dorsal surface of tongue

© 2023 Van Buren I et al. *JAMA Network Open.*

C02.1	Malignant neoplasm of border of tongue
C02.2	Malignant neoplasm of ventral surface of tongue
C02.3	Malig neoplasm of anterior two-thirds of tongue, part unsp
C02.4	Malignant neoplasm of lingual tonsil
C02.8	Malignant neoplasm of overlapping sites of tongue
C02.9	Malignant neoplasm of tongue, unspecified
C03.0	Malignant neoplasm of upper gum
C03.1	Malignant neoplasm of lower gum
C03.9	Malignant neoplasm of gum, unspecified
C04.0	Malignant neoplasm of anterior floor of mouth
C04.1	Malignant neoplasm of lateral floor of mouth
C04.8	Malignant neoplasm of overlapping sites of floor of mouth
C04.9	Malignant neoplasm of floor of mouth, unspecified
C05.0	Malignant neoplasm of hard palate
C05.1	Malignant neoplasm of soft palate
C05.2	Malignant neoplasm of uvula
C05.8	Malignant neoplasm of overlapping sites of palate
C05.9	Malignant neoplasm of palate, unspecified
C06.0	Malignant neoplasm of cheek mucosa
C06.1	Malignant neoplasm of vestibule of mouth
C06.2	Malignant neoplasm of retromolar area
C06.80	Malignant neoplasm of ovrlp sites of unsp parts of mouth
C06.89	Malignant neoplasm of overlapping sites of oth prt mouth
C06.9	Malignant neoplasm of mouth, unspecified
C07	Malignant neoplasm of parotid gland
C08.0	Malignant neoplasm of submandibular gland
C08.1	Malignant neoplasm of sublingual gland
C08.9	Malignant neoplasm of major salivary gland, unspecified
C09.0	Malignant neoplasm of tonsillar fossa
C09.1	Malig neoplasm of tonsillar pillar (anterior) (posterior)
C09.8	Malignant neoplasm of overlapping sites of tonsil
C09.9	Malignant neoplasm of tonsil, unspecified
C10.0	Malignant neoplasm of vallecula
C10.1	Malignant neoplasm of anterior surface of epiglottis
C10.2	Malignant neoplasm of lateral wall of oropharynx
C10.3	Malignant neoplasm of posterior wall of oropharynx
C10.4	Malignant neoplasm of branchial cleft
C10.8	Malignant neoplasm of overlapping sites of oropharynx
C10.9	Malignant neoplasm of oropharynx, unspecified
C11.0	Malignant neoplasm of superior wall of nasopharynx
C11.1	Malignant neoplasm of posterior wall of nasopharynx
C11.2	Malignant neoplasm of lateral wall of nasopharynx
**Code**	**Description**
C11.3	Malignant neoplasm of anterior wall of nasopharynx
C11.8	Malignant neoplasm of overlapping sites of nasopharynx
C11.9	Malignant neoplasm of nasopharynx, unspecified
C12.	Malignant neoplasm of pyriform sinus
C13.0	Malignant neoplasm of postcricoid region
C13.1	Malig neoplasm of aryepiglottic fold, hypopharyngeal aspect
C13.2	Malignant neoplasm of posterior wall of hypopharynx
C13.8	Malignant neoplasm of overlapping sites of hypopharynx
C13.9	Malignant neoplasm of hypopharynx, unspecified
C14.0	Malignant neoplasm of pharynx, unspecified
C14.2	Malignant neoplasm of Waldeyer's ring
C14.8	Malig neoplm of ovrlp sites of lip, oral cavity and pharynx
C32.8	Malignant neoplasm of overlapping sites of larynx
C32.9	Malignant neoplasm of larynx, unspecified

© 2023 Van Buren I et al. *JAMA Network Open.*

eTable 2e. Liver and intrahepatic bile duct cancer ICD codes

Code	Description
155.0	Malignant neoplasm of liver, primary
155.2	Malignant neoplasm of liver, not specified as primary or secondary
C22.0	Liver cell carcinoma
C22.7	Other specified carcinomas of liver
C22.8	Malignant neoplasm of liver, primary, unspecified as to type
C22.9	Malig neoplasm of liver, not specified as primary or sec

eTable 2f. Gastroesophageal ICD codes

Code	Description
150	Malignant neoplasm of cervical esophagus
150.1	Malignant neoplasm of thoracic esophagus
150.2	Malignant neoplasm of abdominal esophagus
150.3	Malignant neoplasm of upper third of esophagus
150.4	Malignant neoplasm of middle third of esophagus
150.5	Malignant neoplasm of lower third of esophagus
150.8	Malignant neoplasm of other specified part of esophagus
150.9	Malignant neoplasm of esophagus unspecified site
151	Malignant neoplasm of cardia
151.1	Malignant neoplasm of pylorus
151.2	Malignant neoplasm of pyloric antrum
151.3	Malignant neoplasm of fundus of stomach
151.4	Malignant neoplasm of body of stomach
151.5	Malignant neoplasm of lesser curvature of stomach unspecified
151.6	Malignant neoplasm of greater curvature of stomach unspecified
151.8	Malignant neoplasm of other specified sites of stomach
151.9	Malignant neoplasm of stomach unspecified site
C15.3	Malignant neoplasm of upper third of esophagus
C15.4	Malignant neoplasm of middle third of esophagus
C15.5	Malignant neoplasm of lower third of esophagus
C15.8	Malignant neoplasm of overlapping sites of esophagus
C15.9	Malignant neoplasm of esophagus, unspecified
C16.0	Malignant neoplasm of cardia
C16.1	Malignant neoplasm of fundus of stomach
C16.2	Malignant neoplasm of body of stomach
C16.3	Malignant neoplasm of pyloric antrum
C16.4	Malignant neoplasm of pylorus
C16.5	Malignant neoplasm of lesser curvature of stomach, unspecified
C16.6	Malignant neoplasm of greater curvature of stomach, unspecified
C16.8	Malignant neoplasm of overlapping sites of stomach
C16.9	Malignant neoplasm of stomach, unspecified

eTable 2g. Colorectal ICD codes

Code	Description
153	Malignant neoplasm of hepatic flexure
153.1	Malignant neoplasm of transverse colon
153.2	Malignant neoplasm of descending colon
153.3	Malignant neoplasm of sigmoid colon
153.4	Malignant neoplasm of cecum
153.5	Malignant neoplasm of appendix vermiformis
153.6	Malignant neoplasm of ascending colon
153.7	Malignant neoplasm of splenic flexure
153.8	Malignant neoplasm of other specified sites of large intestine
153.9	Malignant neoplasm of colon unspecified site
154	Malignant neoplasm of rectosigmoid junction
154.1	Malignant neoplasm of rectum

© 2023 Van Buren I et al. *JAMA Network Open.*

C18.0	Malignant neoplasm of cecum
C18.1	Malignant neoplasm of appendix
C18.2	Malignant neoplasm of ascending colon
C18.3	Malignant neoplasm of hepatic flexure
C18.4	Malignant neoplasm of transverse colon
C18.5	Malignant neoplasm of splenic flexure
C18.6	Malignant neoplasm of descending colon
C18.7	Malignant neoplasm of sigmoid colon
C18.8	Malignant neoplasm of overlapping sites of colon
C18.9	Malignant neoplasm of colon, unspecified
C19	Malignant neoplasm of rectosigmoid junction
C20	Malignant neoplasm of rectum

eTable 2h. Hodgkin's Lymphoma ICD codes

Code	Description
201.00	Hodgkin's paragranuloma, unspecified site, extranodal and solid organ sites
201.01	Hodgkin's paragranuloma, lymph nodes of head, face, and neck
201.02	Hodgkin's paragranuloma, intrathoracic lymph nodes
201.03	Hodgkin's paragranuloma, intra-abdominal lymph nodes
201.04	Hodgkin's paragranuloma, lymph nodes of axilla and upper limb
201.05	Hodgkin's paragranuloma, lymph nodes of inguinal region and lower limb
201.06	Hodgkin's paragranuloma, intrapelvic lymph nodes
201.07	Hodgkin's paragranuloma, spleen
201.08	Hodgkin's paragranuloma, lymph nodes of multiple sites
201.10	Hodgkin's granuloma, unspecified site, extranodal and solid organ sites
201.11	Hodgkin's granuloma, lymph nodes of head, face, and neck
201.12	Hodgkin's granuloma, intrathoracic lymph nodes
201.13	Hodgkin's granuloma, intra-abdominal lymph nodes
201.14	Hodgkin's granuloma, lymph nodes of axilla and upper limb
201.15	Hodgkin's granuloma, lymph nodes of inguinal region and lower limb
201.16	Hodgkin's granuloma, intrapelvic lymph nodes
201.17	Hodgkin's granuloma, spleen
201.18	Hodgkin's granuloma, lymph nodes of multiple sites
201.20	Hodgkin's sarcoma, unspecified site, extranodal and solid organ sites
201.21	Hodgkin's sarcoma, lymph nodes of head, face, and neck
201.22	Hodgkin's sarcoma, intrathoracic lymph nodes
201.23	Hodgkin's sarcoma, intra-abdominal lymph nodes
201.24	Hodgkin's sarcoma, lymph nodes of axilla and upper limb
201.25	Hodgkin's sarcoma, lymph nodes of inguinal region and lower limb
**Code**	**Description**
201.26	Hodgkin's sarcoma, intrapelvic lymph nodes
201.27	Hodgkin's sarcoma, spleen
201.28	Hodgkin's sarcoma, lymph nodes of multiple sites
201.40	Hodgkin's disease, lymphocytic-histiocytic predominance, unspecified site, extranodal and solid organ sites
201.41	Hodgkin's disease, lymphocytic-histiocytic predominance, lymph nodes of head, face, and neck
201.42	Hodgkin's disease, lymphocytic-histiocytic predominance, intrathoracic lymph nodes
201.43	Hodgkin's disease, lymphocytic-histiocytic predominance, intra-abdominal lymph nodes

© 2023 Van Buren I et al. *JAMA Network Open.*

201.44	Hodgkin's disease, lymphocytic-histiocytic predominance, lymph nodes of axilla and upper limb
201.45	Hodgkin's disease, lymphocytic-histiocytic predominance, lymph nodes of inguinal region and lower limb
201.46	Hodgkin's disease, lymphocytic-histiocytic predominance, intrapelvic lymph nodes
201.47	Hodgkin's disease, lymphocytic-histiocytic predominance, spleen
201.48	Hodgkin's disease, lymphocytic-histiocytic predominance, lymph nodes of multiple sites
201.50	Hodgkin's disease, nodular sclerosis, unspecified site, extranodal and solid organ sites
201.51	Hodgkin's disease, nodular sclerosis, lymph nodes of head, face, and neck
201.52	Hodgkin's disease, nodular sclerosis, intrathoracic lymph nodes
201.53	Hodgkin's disease, nodular sclerosis, intra-abdominal lymph nodes
201.54	Hodgkin's disease, nodular sclerosis, lymph nodes of axilla and upper limb
201.55	Hodgkin's disease, nodular sclerosis, lymph nodes of inguinal region and lower limb
201.56	Hodgkin's disease, nodular sclerosis, intrapelvic lymph nodes
201.57	Hodgkin's disease, nodular sclerosis, spleen
201.58	Hodgkin's disease, nodular sclerosis, lymph nodes of multiple sites
201.60	Hodgkin's disease, mixed cellularity, unspecified site, extranodal and solid organ sites
201.61	Hodgkin's disease, mixed cellularity, lymph nodes of head, face, and neck
201.62	Hodgkin's disease, mixed cellularity, intrathoracic lymph nodes
201.63	Hodgkin's disease, mixed cellularity, intra-abdominal lymph nodes
201.64	Hodgkin's disease, mixed cellularity, lymph nodes of axilla and upper limb
201.65	Hodgkin's disease, mixed cellularity, lymph nodes of inguinal region and lower limb
201.66	Hodgkin's disease, mixed cellularity, intrapelvic lymph nodes
201.67	Hodgkin's disease, mixed cellularity, spleen
201.68	Hodgkin's disease, mixed cellularity, lymph nodes of multiple sites
201.70	Hodgkin's disease, lymphocytic depletion, unspecified site, extranodal and solid organ sites
201.71	Hodgkin's disease, lymphocytic depletion, lymph nodes of head, face, and neck
201.72	Hodgkin's disease, lymphocytic depletion, intrathoracic lymph nodes
201.73	Hodgkin's disease, lymphocytic depletion, intra-abdominal lymph nodes
201.74	Hodgkin's disease, lymphocytic depletion, lymph nodes of axilla and upper limb
201.75	Hodgkin's disease, lymphocytic depletion, lymph nodes of inguinal region and lower limb
201.76	Hodgkin's disease, lymphocytic depletion, intrapelvic lymph nodes
201.77	Hodgkin's disease, lymphocytic depletion, spleen
201.78	Hodgkin's disease, lymphocytic depletion, lymph nodes of multiple sites
**Code**	**Description**
201.90	Hodgkin's disease, unspecified type, unspecified site, extranodal and solid organ sites
201.91	Hodgkin's disease, unspecified type, lymph nodes of head, face, and neck
201.92	Hodgkin's disease, unspecified type, intrathoracic lymph nodes
201.93	Hodgkin's disease, unspecified type, intra-abdominal lymph nodes
201.94	Hodgkin's disease, unspecified type, lymph nodes of axilla and upper limb
201.95	Hodgkin's disease, unspecified type, lymph nodes of inguinal region and lower limb
201.96	Hodgkin's disease, unspecified type, intrapelvic lymph nodes

© 2023 Van Buren I et al. *JAMA Network Open.*

201.97	Hodgkin's disease, unspecified type, spleen
201.98	Hodgkin's disease, unspecified type, lymph nodes of multiple sites
C81.00	Nodular lymphocyte predominant Hodgkin lymphoma, unsp site
C81.01	Nodlr lymphocy predom Hdgkn lymph, nodes of head, face, & nk
C81.02	Nodular lymphocy predom Hodgkin lymphoma, intrathorac nodes
C81.03	Nodular lymphocyte predom Hodgkin lymphoma, intra-abd nodes
C81.04	Nodlr lymphocy predom Hdgkn lymph, nodes of axla and upr lmb
C81.05	Nodlr lymphocy predom Hdgkn lymph,nodes of ing rgn & low lmb
C81.06	Nodular lymphocyte predom Hodgkin lymphoma, intrapelv nodes
C81.07	Nodular lymphocyte predominant Hodgkin lymphoma, spleen
C81.08	Nodular lymphocyte predom Hodgkin lymphoma, nodes mult site
C81.09	Nodlr lymphocy predom Hdgkn lymph, extrnod & solid org site
C81.10	Nodular sclerosis Hodgkin lymphoma, unsp site
C81.11	Nodlr scler Hdgkn lymph, nodes of head, face, and neck
C81.12	Nodular sclerosis Hodgkin lymphoma, intrathorac nodes
C81.13	Nodular sclerosis Hodgkin lymphoma, intra-abd nodes
C81.14	Nodlr scler Hdgkn lymph, nodes of axla and upper limb
C81.15	Nodlr scler Hdgkn lymph, nodes of ing rgn and low limb
C81.16	Nodular sclerosis Hodgkin lymphoma, intrapelv nodes
C81.17	Nodular sclerosis Hodgkin lymphoma, spleen
C81.18	Nodular sclerosis Hodgkin lymphoma, nodes mult site
C81.19	Nodlr scler Hdgkn lymph, extrnod and solid organ sites
C81.20	Mixed cellularity Hodgkin lymphoma, unsp site
C81.21	Mix cellular Hdgkn lymph, nodes of head, face, and nk
C81.22	Mixed cellular Hodgkin lymphoma, intrathorac nodes
C81.23	Mixed cellular Hodgkin lymphoma, intra-abd nodes
C81.24	Mix cellular Hdgkn lymph, nodes of axla and upper limb
C81.25	Mix cellular Hdgkn lymph, nodes of ing rgn and low lmb
C81.26	Mixed cellular Hodgkin lymphoma, intrapelv nodes
C81.27	Mixed cellularity Hodgkin lymphoma, spleen
C81.28	Mixed cellular Hodgkin lymphoma, nodes mult site
C81.29	Mix cellular Hdgkn lymph, extrnod and solid org sites
C81.30	Lymphocyte depleted Hodgkin lymphoma, unsp site
C81.31	Lymphocy deplet Hdgkn lymph, nodes of head, face, & nk
C81.32	Lymphocy depleted Hodgkin lymphoma, intrathorac nodes
C81.33	Lymphocy depleted Hodgkin lymphoma, intra-abd nodes
C81.34	Lymphocy deplet Hdgkn lymph, nodes of axla and upr lmb
C81.35	Lymphocy deplet Hdgkn lymph,nodes of ing rgn & low lmb
C81.36	Lymphocy depleted Hodgkin lymphoma, intrapelv nodes
**Code**	**Description**
C81.37	Lymphocyte depleted Hodgkin lymphoma, spleen
C81.38	Lymphocy depleted Hodgkin lymphoma, nodes mult site
C81.39	Lymphocy deplet Hdgkn lymph, extrnod & solid org site
C81.40	Lymphocyte-rich Hodgkin lymphoma, unspecified site
C81.41	Lymp-rich Hodgkin lymph, nodes of head, face, and neck
C81.42	Lymp-rich Hodgkin lymphoma, intrathorac nodes
C81.43	Lymp-rich Hodgkin lymphoma, intra-abd lymph nodes

© 2023 Van Buren I et al. *JAMA Network Open.*

C81.44	Lymp-rich Hdgkn lymph, nodes of axilla and upper limb
C81.45	Lymp-rich Hdgkn lymph, nodes of ing rgn and lower limb
C81.46	Lymp-rich Hodgkin lymphoma, intrapelv lymph nodes
C81.47	Lymphocyte-rich Hodgkin lymphoma, spleen
C81.48	Lymp-rich Hodgkin lymphoma, lymph nodes mult site
C81.49	Lymp-rich Hodgkin lymph, extrnod and solid organ sites
C81.70	Other Hodgkin lymphoma, unspecified site
C81.71	Oth Hodgkin lymphoma, nodes of head, face, and neck
C81.72	Other Hodgkin lymphoma, intrathoracic lymph nodes
C81.73	Oth Hodgkin lymphoma, intra-abdominal lymph nodes
C81.74	Oth Hodgkin lymphoma, nodes of axilla and upper limb
C81.75	Oth Hodgkin lymph, nodes of ing region and lower limb
C81.76	Other Hodgkin lymphoma, intrapelvic lymph nodes
C81.77	Other Hodgkin lymphoma, spleen
C81.78	Oth Hodgkin lymphoma, lymph nodes mult site
C81.79	Oth Hodgkin lymphoma, extrnod and solid organ sites
C81.90	Hodgkin lymphoma, unspecified, unspecified site
C81.91	Hodgkin lymphoma, unsp, lymph nodes of head, face, and neck
C81.92	Hodgkin lymphoma, unspecified, intrathoracic lymph nodes
C81.93	Hodgkin lymphoma, unspecified, intra-abdominal lymph nodes
C81.94	Hodgkin lymphoma, unsp, lymph nodes of axilla and upper limb
C81.95	Hodgkin lymphoma, unsp, nodes of ing region and lower limb
C81.96	Hodgkin lymphoma, unspecified, intrapelvic lymph nodes
C81.97	Hodgkin lymphoma, unspecified, spleen
C81.98	Hodgkin lymphoma, unspecified, lymph nodes of multiple sites
C81.99	Hodgkin lymphoma, unsp, extranodal and solid organ sites

eTable 2i. Merkel cell carcinoma ICD codes

Code	Description
209.36	Merkel cell carcinoma of other sites
C4A.9	Merkel cell carcinoma, unspecified

eTable 2j. Mesothelioma ICD codes

Code	Description
163.9	Malignant neoplasm of pleura, unspecified
C45.0	Mesothelioma of pleura
C45.1	Mesothelioma of peritoneum
C45.2	Mesothelioma of pericardium
C45.7	Mesothelioma of other sites
C45.9	Mesothelioma, unspecified

eTable 2k. Anal cancers ICD codes

Code	Description
154.2	Malignant neoplasm of anal canal
154.3	Malignant neoplasm of anus, unspecified site
**Code**	**Description**
154.8	Malignant neoplasm of other sites of rectum, rectosigmoid junction, and anus
C21.0	Malignant neoplasm of anus, unspecified
C21.1	Malignant neoplasm of anal canal
C21.2	Malignant neoplasm of cloacogenic zone
C21.8	Malig neoplasm of ovrlp sites of rectum, anus and anal canal

© 2023 Van Buren I et al. *JAMA Network Open.*

eTable 2L. Breast cancer ICD codes

Code	Description
174.0	Malignant neoplasm of nipple and areola of female breast
174.1	Malignant neoplasm of central portion of female breast
174.2	Malignant neoplasm of upper-inner quadrant of female breast
174.3	Malignant neoplasm of lower-inner quadrant of female breast
174.4	Malignant neoplasm of upper-outer quadrant of female breast
174.5	Malignant neoplasm of lower-outer quadrant of female breast
174.6	Malignant neoplasm of axillary tail of female breast
174.8	Malignant neoplasm of other specified sites of female breast
174.9	Malignant neoplasm of breast (female), unspecified
175.0	Malignant neoplasm of nipple and areola of male breast
175.9	Malignant neoplasm of other and unspecified sites of male breast
C50.011	Malignant neoplasm of nipple and areola, right female breast
C50.012	Malignant neoplasm of nipple and areola, left female breast
C50.019	Malignant neoplasm of nipple and areola, unsp female breast
C50.021	Malignant neoplasm of nipple and areola, right male breast
C50.022	Malignant neoplasm of nipple and areola, left male breast
C50.029	Malignant neoplasm of nipple and areola, unsp male breast
C50.111	Malignant neoplasm of central portion of right female breast
C50.112	Malignant neoplasm of central portion of left female breast
C50.119	Malignant neoplasm of central portion of unsp female breast
C50.121	Malignant neoplasm of central portion of right male breast
C50.122	Malignant neoplasm of central portion of left male breast
C50.129	Malignant neoplasm of central portion of unsp male breast
C50.211	Malig neoplm of upper-inner quadrant of right female breast
C50.212	Malig neoplasm of upper-inner quadrant of left female breast
C50.219	Malig neoplasm of upper-inner quadrant of unsp female breast
C50.221	Malig neoplasm of upper-inner quadrant of right male breast
C50.222	Malig neoplasm of upper-inner quadrant of left male breast
C50.229	Malig neoplasm of upper-inner quadrant of unsp male breast
C50.311	Malig neoplm of lower-inner quadrant of right female breast
C50.312	Malig neoplasm of lower-inner quadrant of left female breast
C50.319	Malig neoplasm of lower-inner quadrant of unsp female breast
C50.321	Malig neoplasm of lower-inner quadrant of right male breast
C50.322	Malig neoplasm of lower-inner quadrant of left male breast
C50.329	Malig neoplasm of lower-inner quadrant of unsp male breast
C50.411	Malig neoplm of upper-outer quadrant of right female breast
C50.412	Malig neoplasm of upper-outer quadrant of left female breast
C50.419	Malig neoplasm of upper-outer quadrant of unsp female breast
C50.421	Malig neoplasm of upper-outer quadrant of right male breast
C50.422	Malig neoplasm of upper-outer quadrant of left male breast
**Code**	**Description**
C50.429	Malig neoplasm of upper-outer quadrant of unsp male breast
C50.511	Malig neoplm of lower-outer quadrant of right female breast
C50.512	Malig neoplasm of lower-outer quadrant of left female breast
C50.519	Malig neoplasm of lower-outer quadrant of unsp female breast

© 2023 Van Buren I et al. *JAMA Network Open.*

C50.521	Malig neoplasm of lower-outer quadrant of right male breast
C50.522	Malig neoplasm of lower-outer quadrant of left male breast
C50.529	Malig neoplasm of lower-outer quadrant of unsp male breast
C50.611	Malignant neoplasm of axillary tail of right female breast
C50.612	Malignant neoplasm of axillary tail of left female breast
C50.619	Malignant neoplasm of axillary tail of unsp female breast
C50.621	Malignant neoplasm of axillary tail of right male breast
C50.622	Malignant neoplasm of axillary tail of left male breast
C50.629	Malignant neoplasm of axillary tail of unsp male breast
C50.811	Malignant neoplasm of ovrlp sites of right female breast
C50.812	Malignant neoplasm of ovrlp sites of left female breast
C50.819	Malignant neoplasm of ovrlp sites of unsp female breast
C50.821	Malignant neoplasm of overlapping sites of right male breast
C50.822	Malignant neoplasm of overlapping sites of left male breast
C50.829	Malignant neoplasm of overlapping sites of unsp male breast
C50.911	Malignant neoplasm of unsp site of right female breast
C50.912	Malignant neoplasm of unspecified site of left female breast
C50.919	Malignant neoplasm of unsp site of unspecified female breast
C50.921	Malignant neoplasm of unspecified site of right male breast
C50.922	Malignant neoplasm of unspecified site of left male breast
C50.929	Malignant neoplasm of unsp site of unspecified male breast

eTable 2m. All metastasis ICD codes

Code	Description
196.0	Secondary and unspecified malignant neoplasm of lymph nodes of head, face, and neck
196.1	Secondary and unspecified malignant neoplasm of intrathoracic lymph nodes
196.2	Secondary and unspecified malignant neoplasm of intra-abdominal lymph nodes
196.3	Secondary and unspecified malignant neoplasm of lymph nodes of axilla and upper limb
196.5	Secondary and unspecified malignant neoplasm of lymph nodes of inguinal region and lower limb
196.6	Secondary and unspecified malignant neoplasm of intrapelvic lymph nodes
196.8	Secondary and unspecified malignant neoplasm of lymph nodes of multiple sites
196.9	Secondary and unspecified malignant neoplasm of lymph nodes, site unspecified
197.0	Secondary malignant neoplasm of lung
197.1	Secondary malignant neoplasm of mediastinum
197.2	Secondary malignant neoplasm of pleura
197.3	Secondary malignant neoplasm of other respiratory organs
197.4	Secondary malignant neoplasm of small intestine including duodenum
197.5	Secondary malignant neoplasm of large intestine and rectum
197.6	Secondary malignant neoplasm of retroperitoneum and peritoneum
197.7	Malignant neoplasm of liver, secondary
197.8	Secondary malignant neoplasm of other digestive organs and spleen
198.0	Secondary malignant neoplasm of kidney
198.1	Secondary malignant neoplasm of other urinary organs
198.2	Secondary malignant neoplasm of skin
198.3	Secondary malignant neoplasm of brain and spinal cord
198.4	Secondary malignant neoplasm of other parts of nervous system
198.5	Secondary malignant neoplasm of bone and bone marrow
**Code**	**Description**
198.6	Secondary malignant neoplasm of ovary
198.7	Secondary malignant neoplasm of adrenal gland
198.81	Secondary malignant neoplasm of breast
198.82	Secondary malignant neoplasm of genital organs

© 2023 Van Buren I et al. *JAMA Network Open.*

198.89	Secondary malignant neoplasm of other specified sites
C77.0	Sec and unsp malig neoplasm of nodes of head, face and neck
C77.1	Secondary and unsp malignant neoplasm of intrathorac nodes
C77.2	Secondary and unsp malignant neoplasm of intra-abd nodes
C77.3	Sec and unsp malig neoplasm of axilla and upper limb nodes
C77.4	Sec and unsp malig neoplasm of inguinal and lower limb nodes
C77.5	Secondary and unsp malignant neoplasm of intrapelv nodes
C77.8	Sec and unsp malig neoplasm of nodes of multiple regions
C77.9	Secondary and unsp malignant neoplasm of lymph node, unsp
C78.00	Secondary malignant neoplasm of unspecified lung
C78.01	Secondary malignant neoplasm of right lung
C78.02	Secondary malignant neoplasm of left lung
C78.1	Secondary malignant neoplasm of mediastinum
C78.2	Secondary malignant neoplasm of pleura
C78.30	Secondary malignant neoplasm of unsp respiratory organ
C78.39	Secondary malignant neoplasm of other respiratory organs
C78.4	Secondary malignant neoplasm of small intestine
C78.5	Secondary malignant neoplasm of large intestine and rectum
C78.6	Secondary malignant neoplasm of retroperiton and peritoneum
C78.7	Secondary malig neoplasm of liver and intrahepatic bile duct
C78.80	Secondary malignant neoplasm of unspecified digestive organ
C78.89	Secondary malignant neoplasm of other digestive organs
C79.00	Secondary malignant neoplasm of unsp kidney and renal pelvis
C79.01	Secondary malignant neoplasm of r kidney and renal pelvis
C79.02	Secondary malignant neoplasm of left kidney and renal pelvis
C79.10	Secondary malignant neoplasm of unspecified urinary organs
C79.11	Secondary malignant neoplasm of bladder
C79.19	Secondary malignant neoplasm of other urinary organs
C79.2	Secondary malignant neoplasm of skin
C79.31	Secondary malignant neoplasm of brain
C79.32	Secondary malignant neoplasm of cerebral meninges
C79.40	Secondary malignant neoplasm of unsp part of nervous system
C79.49	Secondary malignant neoplasm of oth parts of nervous system
C79.51	Secondary malignant neoplasm of bone
C79.52	Secondary malignant neoplasm of bone marrow
C79.60	Secondary malignant neoplasm of unspecified ovary
C79.61	Secondary malignant neoplasm of right ovary
C79.62	Secondary malignant neoplasm of left ovary
C79.63	Secondary malignant neoplasm of bilateral ovaries
C79.70	Secondary malignant neoplasm of unspecified adrenal gland
C79.71	Secondary malignant neoplasm of right adrenal gland
C79.72	Secondary malignant neoplasm of left adrenal gland
C79.81	Secondary malignant neoplasm of breast
C79.82	Secondary malignant neoplasm of genital organs
C79.89	Secondary malignant neoplasm of other specified sites
C79.9	Secondary malignant neoplasm of unspecified site

eTable 2n. Lymph metastasis ICD codes

196.0	Secondary and unspecified malignant neoplasm of lymph nodes of head, face, and neck
196.1	Secondary and unspecified malignant neoplasm of intrathoracic lymph nodes
196.2	Secondary and unspecified malignant neoplasm of intra-abdominal lymph nodes
**Code**	**Description**
196.3	Secondary and unspecified malignant neoplasm of lymph nodes of axilla and upper limb
196.5	Secondary and unspecified malignant neoplasm of lymph nodes of inguinal region and lower limb
196.6	Secondary and unspecified malignant neoplasm of intrapelvic lymph nodes
196.8	Secondary and unspecified malignant neoplasm of lymph nodes of multiple sites

© 2023 Van Buren I et al. *JAMA Network Open.*

196.9	Secondary and unspecified malignant neoplasm of lymph nodes, site unspecified
C77.0	Sec and unsp malig neoplasm of nodes of head, face and neck
C77.1	Secondary and unsp malignant neoplasm of intrathorac nodes
C77.2	Secondary and unsp malignant neoplasm of intra-abd nodes
C77.3	Sec and unsp malig neoplasm of axilla and upper limb nodes
C77.4	Sec and unsp malig neoplasm of inguinal and lower limb nodes
C77.5	Secondary and unsp malignant neoplasm of intrapelv nodes
C77.8	Sec and unsp malig neoplasm of nodes of multiple regions
C77.9	Secondary and unsp malignant neoplasm of lymph node, unsp

eTable 2o. CNS metastasis ICD codes

Code	Description
198.3	Secondary malignant neoplasm of brain and spinal cord
198.4	Secondary malignant neoplasm of other parts of nervous system
C79.31	Secondary malignant neoplasm of brain
C79.32	Secondary malignant neoplasm of cerebral meninges
C79.40	Secondary malignant neoplasm of unsp part of nervous system
C79.49	Secondary malignant neoplasm of oth parts of nervous system

© 2023 Van Buren I et al. *JAMA Network Open.*

eTable 3. Characteristics of the Veteran cohort in steroid subgroups

Characteristic	S:irAE n = 5004	S:other n = 7217	Baseline use n = 3275	Dexamethasone only n = 4045	Prophylaxis n = 1512	Isolated dose n = 2028
Age at first ICI tx, median (MAD; range)	70 (7.4; 20-97)	70 (5.9; 25-98)	69 (5.9; 29-97)	69 (5.9; 25-97)	70 (5.9; 33-97)	70 (5.9; 27-98)
Male, *n* (%)	4874 (97.4)	6956 (96.4)	3132 (95.6)	3906 (96.6)	1460 (96.6)	1955 (96.4)
Number of ICI treatments, mean (SD; range)	14.1 (15.3; 1-139)	10.0 (11.7; 1-140)	9.7 (11.6; 1-124)	8.8 (10.0; 1-107)	10.7 (12.4; 1-115)	10.1 (13.5; 1-140)
CCI, mean (SD; range)	12.8 (3.9; 2-30)	12.7 (3.8; 2-29)	12.8 (3.7; 2-28)	12.7 (3.8; 2-29)	12.7 (3.9; 2-25)	12.6 (3.9; 2-25)
irAE ICD present, *n* (%)	2286 (45.6)	1862 (25.8)	968 (29.6)	813 (20.1)	315 (20.8)	440 (21.7)
**%, N**						
Current/former smoker	58.4% (2926)	66.2% (4777)	66.5% (2177)	67.4% (2727)	70% (1059)	67% (1358)
Never smoker	17.3% (870)	16.7% (1207)	15.4% (504)	16.6% (672)	15.8% (239)	20.3% (411)
**Primary Cancer**						
Bronchus/lung	53.4% (2675)	65.8% (4752)	74.1% (2427)	66.8% (2701)	66.8% (1010)	53.1% (1077)
Urinary tract	15.5% (779)	11% (797)	7.6% (250)	10.5% (425)	10.3% (155)	16% (324)
Melanoma	15.2% (764)	7.8% (560)	4.9% (162)	7.2% (293)	8.9% (134)	11.8% (239)
Head and neck	5.7% (286)	6.1% (442)	4.8% (157)	6.9% (278)	5.4% (81)	6.7% (135)
Liver	5.2% (263)	3.1% (225)	1.5% (48)	2.3% (92)	4.2% (63)	5.5% (112)
Gastroesophageal	1.5% (79)	2.7% (193)	3.3% (108)	3.0% (123)	1.7% (25)	2.7% (54)
Colorectal	0.7% (40)	1.3% (91)	1.6% (53)	1.3% (52)	0.1% (1)	1.2% (25)
Squamous of skin	0.4% (22)	0.4% (29)	0.3% (9)	0.4% (14)	0.3% (5)	0.9% (18)
Mesothelioma	0.4% (25	0.5% (33)	0.3% (11)	0.5% (19)	0.5% (8)	0.6% (13)
Merkel	0.3% (19)	0.5% (33)	0.5% (17)	0.3% (11)	0.5% (8)	0.7% (15)
Hodgkin	0.5% (28)	0.4% (27)	0.4% (13)	0.2% (7)	0.5% (7)	0.4% (8)
Anal	0.3% (17)	0.2% (12)	0.1% (4)	0.2% (9)	0.1% (1)	0.1% (2)
Breast	0.1% (7)	0.3% (23)	0.5% (16)	0.5% (21)	0.2% (3)	0.3% (6)
**Metastasis**						
Any metastases	83.1% (4163)	84.1% (6067)	86% (2817)	86.6% (3501)	78.4% (1185)	78.3% (1588)
Lymph node metastases	45% (2253)	41.3% (2984)	42.7% (1397)	39.1% (1580)	38.5% (582)	36.9% (749)
CNS metastases	17.2% (863)	27% (1945)	30.6% (1003)	33.6% (1360)	17.5% (264)	18% (365)

S:irAE, steroid use pattern suggestive of irAEs; S:other, steroids for non-irAE associated indications; CCI, Charlson Comorbidity Index; MAD, median absolute deviation, SD, standard deviation

© 2023 Van Buren I et al. *JAMA Network Open.*

eTable 4. Primary cancer diagnoses and metastasis data of the entire VA cohort

Primary Cancer	Total Pts (n = 20163)	Any metastases (n = 15885)	Lymph Met (n = 7921)	CNS Met (n = 3741)
**% (N)**				
Bronchus/lung	10992	78.8% (8662)	39.8% (4371)	22.5% (2470)
Urinary tract	2953	85.6% (2528)	36.0% (1063)	11.9% (352)
Melanoma	2349	86.5% (2033)	47.1% (1107)	28.8% (676)
Head and neck	1331	81.2% (1081)	50.6% (674)	8.8% (117)
Liver	1213	52.3% (634)	15.0% (182)	2.6% (32)
Gastroesophageal	521	78.1% (407)	43.0% (224)	9.0% (47)
Colorectal	278	79.9% (222)	37.8% (105)	5.4% (15)
Squamous of skin	132	68.9% (91)	47.0% (62)	3.8% (5)
Mesothelioma	98	53.1% (52)	21.4% (21)	2.0% (2)
Merkel	96	71.9% (69)	56.3% (54)	11.5% (11)
Hodgkin	94	29.8% (28)	13.8% (13)	2.1% (2)
Anal	61	82.0% (50)	49.2% (30)	8.2% (5)
Breast	45	62.2% (28)	33.3% (15)	15.9% (7)

eTable 5. Charlson Score Calculation

Condition	Scoring
**Myocardial infarction**	+1
**Congestive heart failure**	+1
**Peripheral vascular disease**	+1
**Cerebrovascular disease**	+1
**Dementia**	+1
**Chronic pulmonary disease**	+1
**Rheumatologic disease**	+1
**Peptic ulcer disease**	+1
**Mild liver disease**	+1
**Diabetes without chronic complication**	+1
**Diabetes with chronic complication**	+2
**Hemiplegia or paraplegia**	+2
**Renal disease**	+2
**Any malignancy, including leukemia and lymphoma**	+2
**Moderate or severe liver disease**	+3
**Metastatic solid tumor**	+6
**AIDS/HIV**	+6
**Age ≥ 80**	+4
**Age 70-79**	+3
**Age 60-69**	+2
**Age 50-59**	+1

© 2023 Van Buren I et al. *JAMA Network Open.*

eTable 6. irAE types in patients with irAE related ICD codes (+irAE ICD)

Organ system	Overall n = 5461	No steroids n = 1313	Any systemic steroid n = 4148	p-value
% (N)				
GI	34.2% (1865)	28.8% (378)	35.8% (1487)	<0.001
Endocrine	27.8% (1518)	29.3% (385)	27.3% (1133)	0.16
Dermatologic	18.3% (1001)	21.9% (287)	17.2% (714)	<0.001
Pulmonary	7% (382)	1.6% (21)	8.7% (361)	<0.001
Hepatic	6.8% (369)	9.7% (127)	5.8% (242)	<0.001
Renal	5% (274)	7.5% (99)	4.2% (175)	<0.001
Neurologic	0.6% (35)	0.8% (11)	0.6% (24)	0.3
Eye	0.2% (10)	0.2% (3)	0.2% (7)	0.66
Pancreatic	0.1% (7)	0.2% (2)	0.1% (5)	0.28

Adjusted significance threshold p < 0.006.

eFigure 1. Secondary metastases and irAE diagnosis on survival

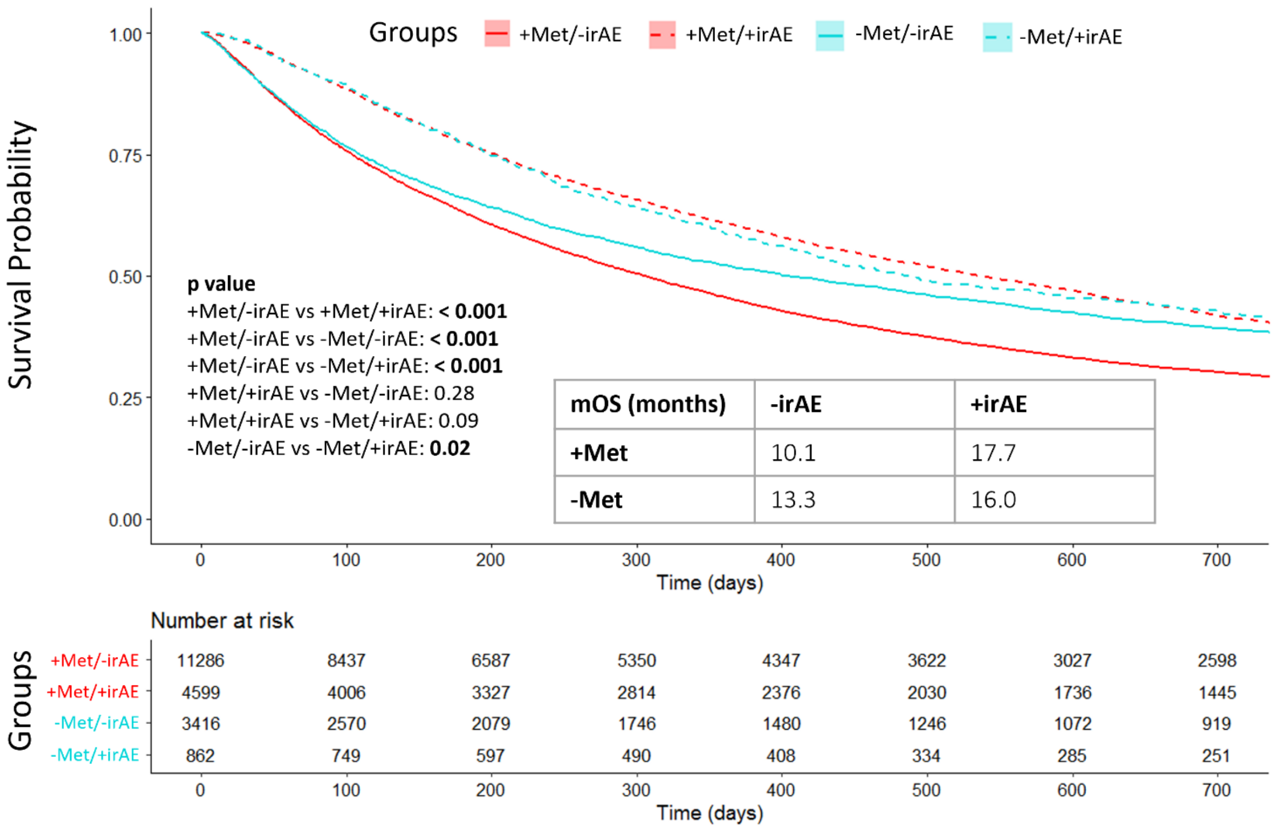

Kaplan-Meier curve showing overall survival in Veterans cohort stratified by the presence of secondary metastases and irAE ICD codes. Survival probability shown on y-axis over time (in days) on x-axis. Bottom table demonstrates remaining number of patients at risk over 8 time points. Groups are stratified by the presence (red) or absence (blue) of secondary metastases, as well as by the presence (dashed line) or absence (solid line) of irAE ICD codes. Median overall survival in months for each group shown under figure key. Adjusted significance threshold p < 0.008.

© 2023 Van Buren I et al. *JAMA Network Open.*

eFigure 2. irAE diagnosis and survival across cancer types

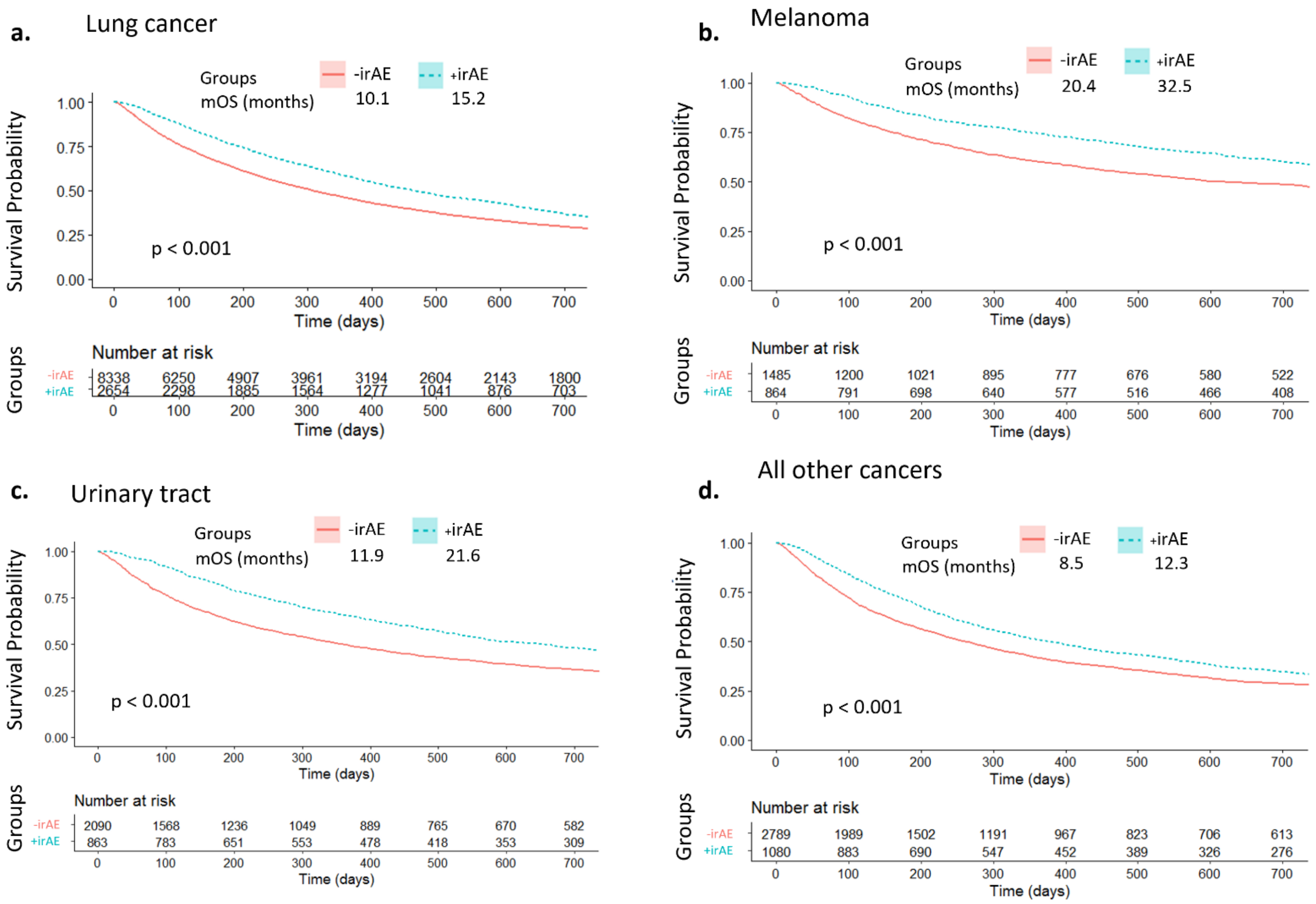

Kaplan-Meier (KM) curves showing overall survival in patients across cancer types without irAE related ICD codes (red) and irAE related ICD codes (blue). Survival probability shown on y-axis over time (in days) on x-axis. Table below graphs demonstrate remaining number of patients at risk over 8 time points. Median overall survival in months for each group shown under figure key. **a.** Lung cancer **b.** Melanoma **c.** Urinary tract cancer **d.** All other remaining cancers.

© 2023 Van Buren I et al. *JAMA Network Open.*

eFigure 3. Secondary metastases and irAE diagnosis on survival across cancer types

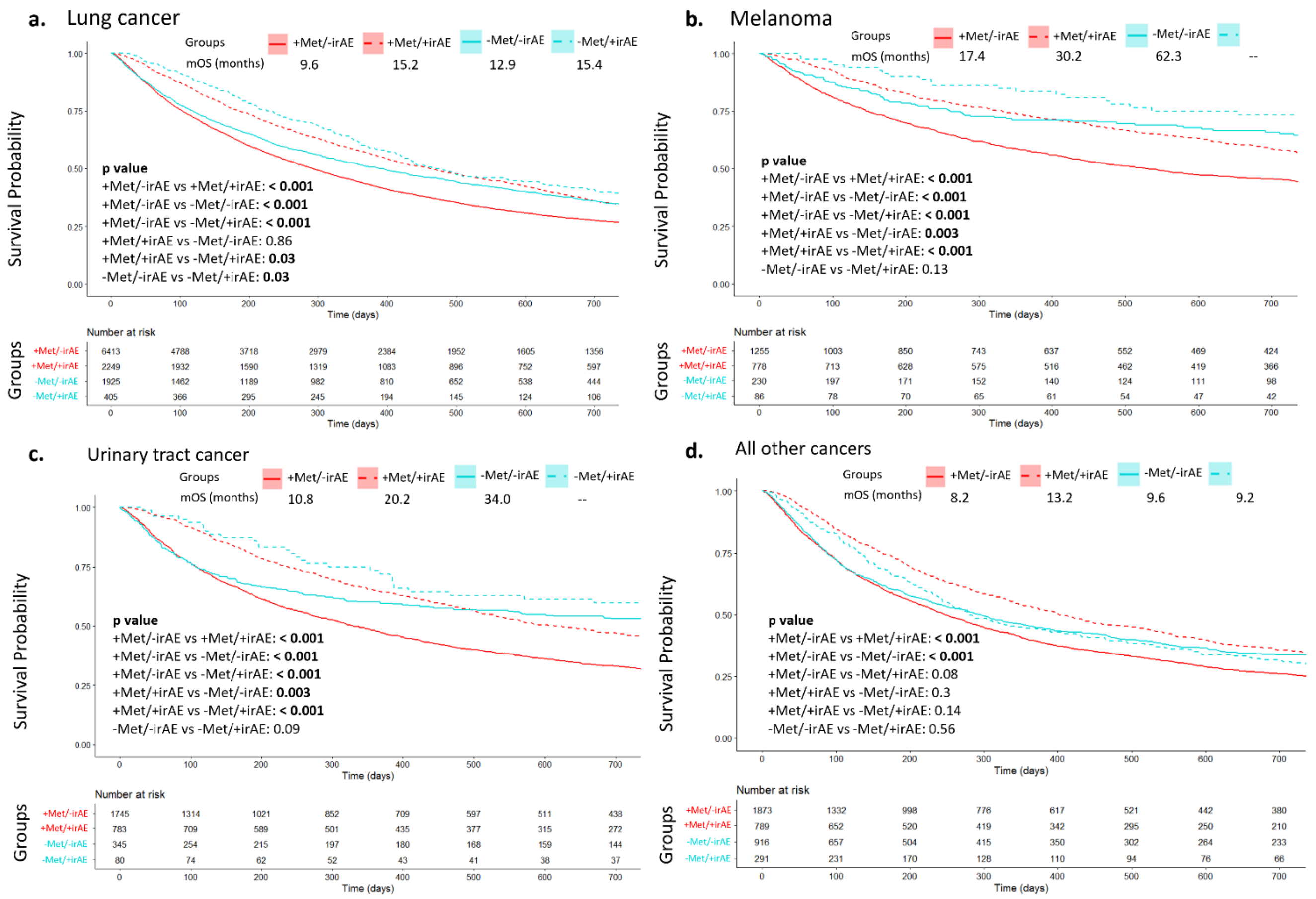

Kaplan-Meier curves showing overall survival in patients across cancer types with presence (red) or absence (blue) of secondary metastases, as well as the presence (dashed line) or absence (solid line) of irAE ICD codes. Survival probability shown on y-axis over time (in days) on x-axis. Table below graphs demonstrate remaining number of patients at risk over 8 time points. Median overall survival in months for each group shown under figure key. **a.** Lung cancer **b.** Melanoma **c.** Urinary tract cancer **d.** All other remaining cancers. Adjusted significance threshold p < 0.008.

eFigure 4. irAE type and survival

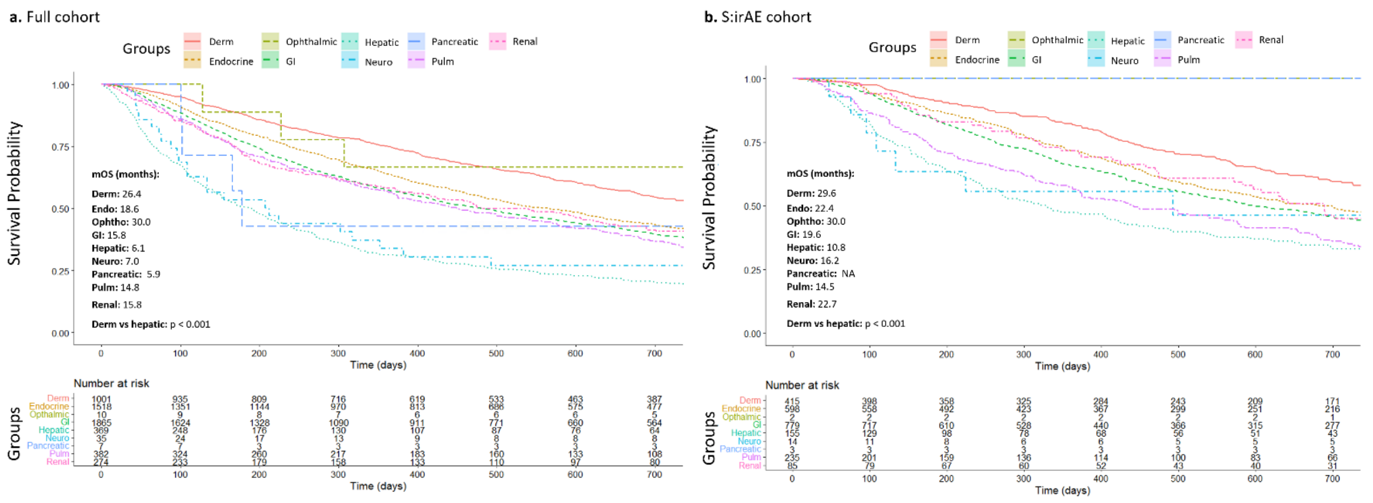

Kaplan-Meier curves showing association between irAE type with overall survival. Survival probability shown on y-axis over time (in days) on x-axis. Table below graphs demonstrate remaining number of patients at risk over 8 time points. Median overall survival (mOS) in months listed. **a.** Full cohort **b.** Patients receiving steroids for irAE management (S:irAE). Adjusted significance threshold p < 0.001.

© 2023 Van Buren I et al. *JAMA Network Open.*

eFigure 5. Steroid use and presence of metastases on survival

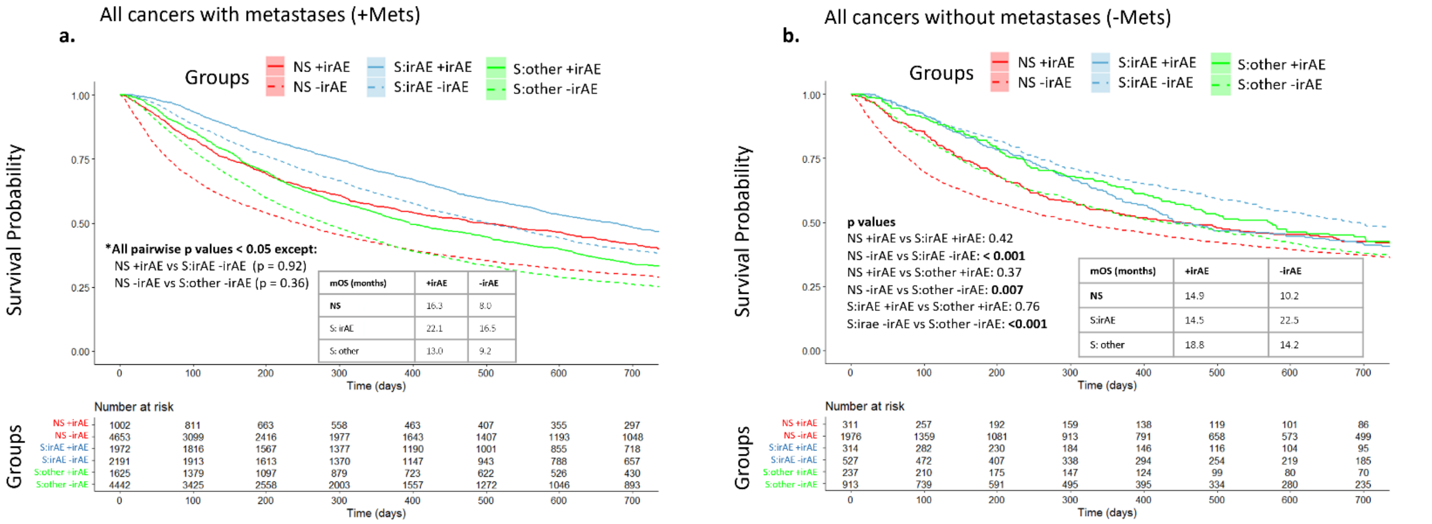

Kaplan-Meier curves showing association between steroid use with overall survival in patients with and without secondary metastases. Survival probability shown on y-axis over time (in days) on x-axis. Table below graphs demonstrate remaining number of patients at risk over 8 time points. No steroid (NS) group is represented in red while patients who received steroids for irAE (S:irAE) vs non-irAE (S:other) are represented in blue and green, respectively. +/- irAE ICD codes represented by solid and dashed lines, respectively. Median OS in months listed in associated tables. Adjusted significance threshold p < 0.003. **a.** Patients with secondary metastases. **b.** Patients without secondary metastases.

eFigure 6. Steroid use and presence of metastases on survival in lung cancer patients

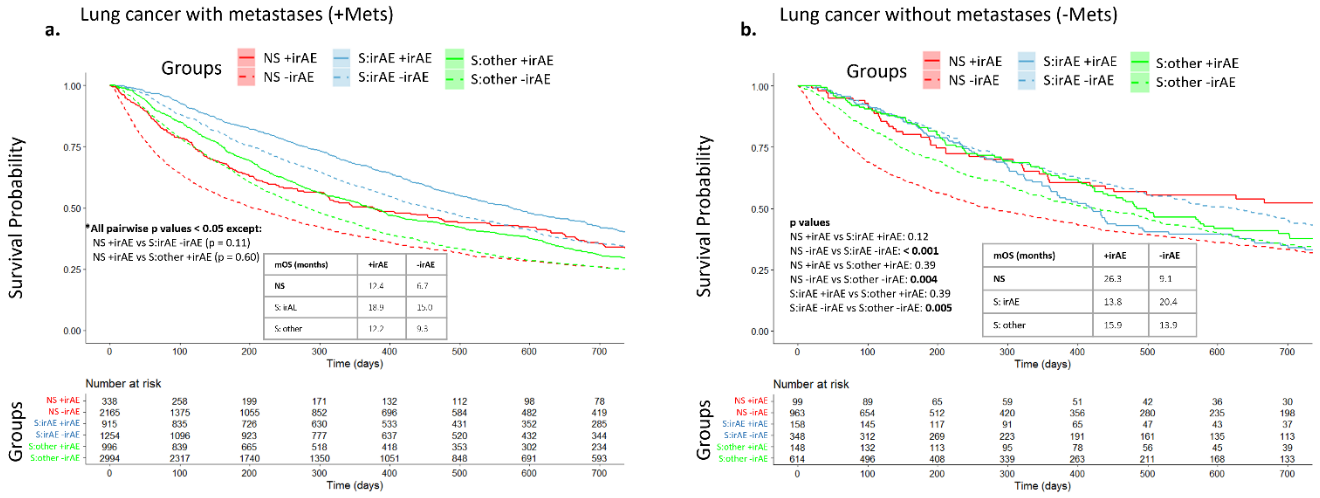

Kaplan-Meier curves showing association between steroid use with overall survival in lung cancer patients with and without secondary metastases. Survival probability shown on y-axis over time (in days) on x-axis. Table below graphs demonstrate remaining number of patients at risk over 8 time points. No steroid (NS) group is represented in red while patients who received steroids for irAE (S:irAE) vs non-irAE (S:other) are represented in blue and green, respectively. +/- irAE ICD codes represented by solid and dashed lines, respectively. Median OS in months listed in associated tables. Adjusted significance threshold p < 0.003. **a.** Lung cancer patients with secondary metastases. **b.** Lung cancer patients without secondary metastases.

© 2023 Van Buren I et al. *JAMA Network Open.*

eFigure 7. Steroid use and presence of metastases on survival in melanoma patients

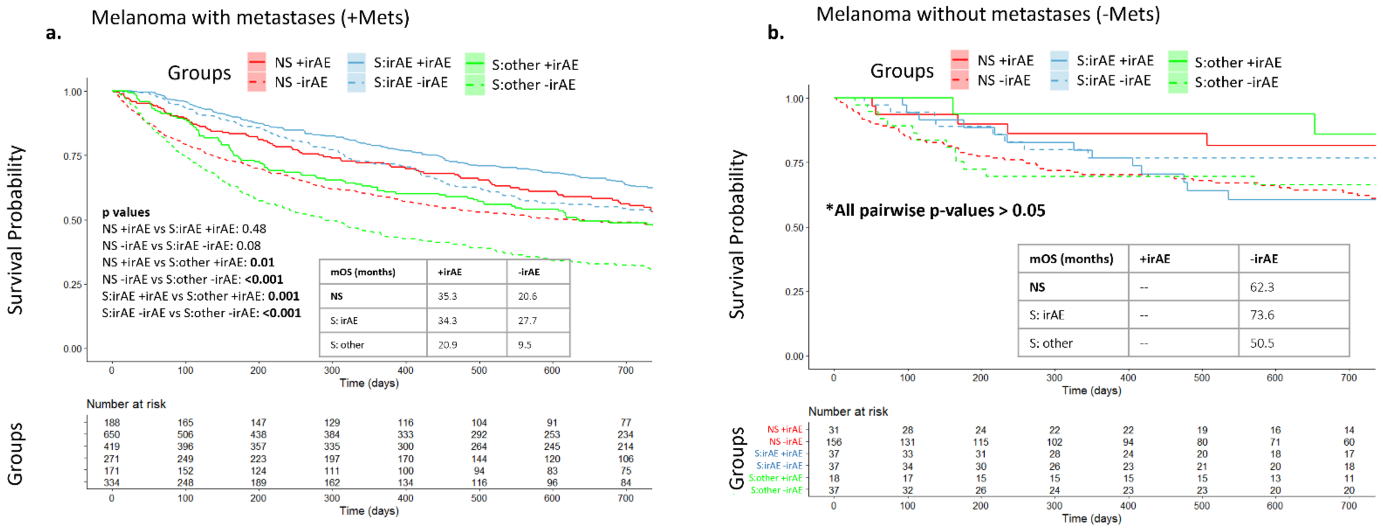

Kaplan-Meier curves showing association between steroid use with overall survival in melanoma patients with and without secondary metastases. Survival probability shown on y-axis over time (in days) on x-axis. Table below graphs demonstrate remaining number of patients at risk over 8 time points. No steroid (NS) group is represented in red while patients who received steroids for irAE (S:irAE) vs non-irAE (S:other) are represented in blue and green, respectively. +/- irAE ICD codes represented by solid and dashed lines, respectively. Median OS in months listed in associated tables. Adjusted significance threshold p < 0.003. **a.** Melanoma patients with secondary metastases. **b.** Melanoma patients without secondary metastases.

eFigure 8. Steroid use and presence of metastases on survival in urinary cancer patients

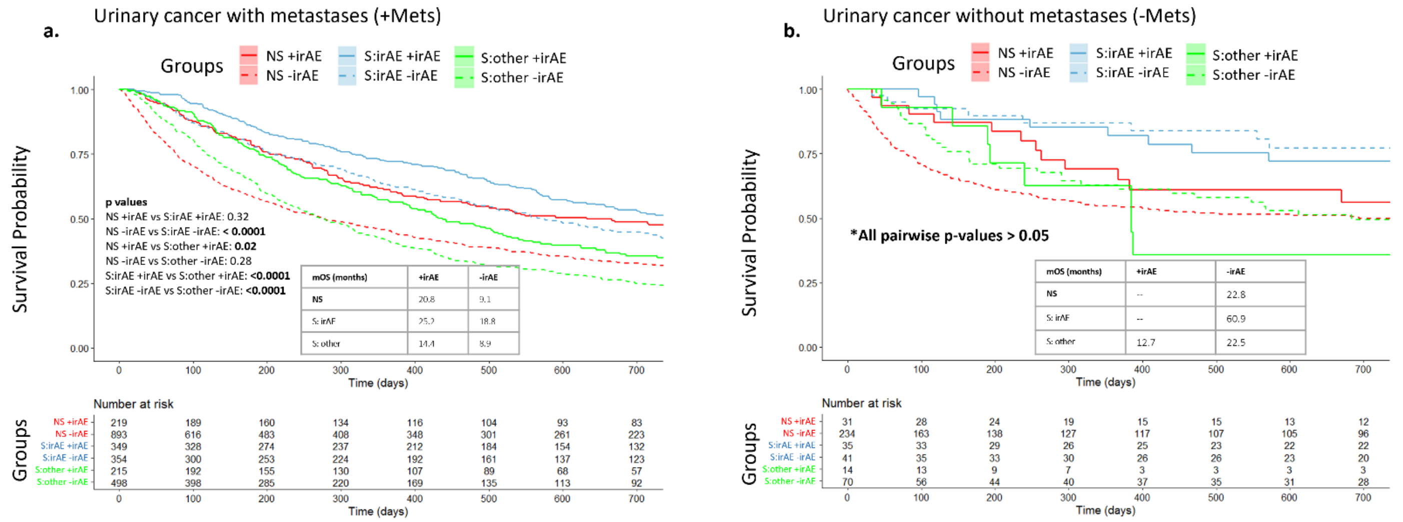

Kaplan-Meier curves showing association between steroid use with overall survival in urinary tract cancer patients with and without secondary metastases. Survival probability shown on y-axis over time (in days) on x-axis. Table below graphs demonstrate remaining number of patients at risk over 8 time points. No steroid (NS) group is represented in red while patients who received steroids for irAE (S:irAE) vs non-irAE (S:other) are represented in blue and green, respectively. +/- irAE ICD codes represented by solid and dashed lines, respectively. Median OS in months listed in associated tables. Adjusted significance threshold p < 0.003. **a.** Urinary tract cancer patients with secondary metastases. **b.** Urinary tract cancer patients without secondary metastases.

© 2023 Van Buren I et al. *JAMA Network Open.*

eFigure 9. Steroid use and presence of metastases on survival in patients with all other cancers

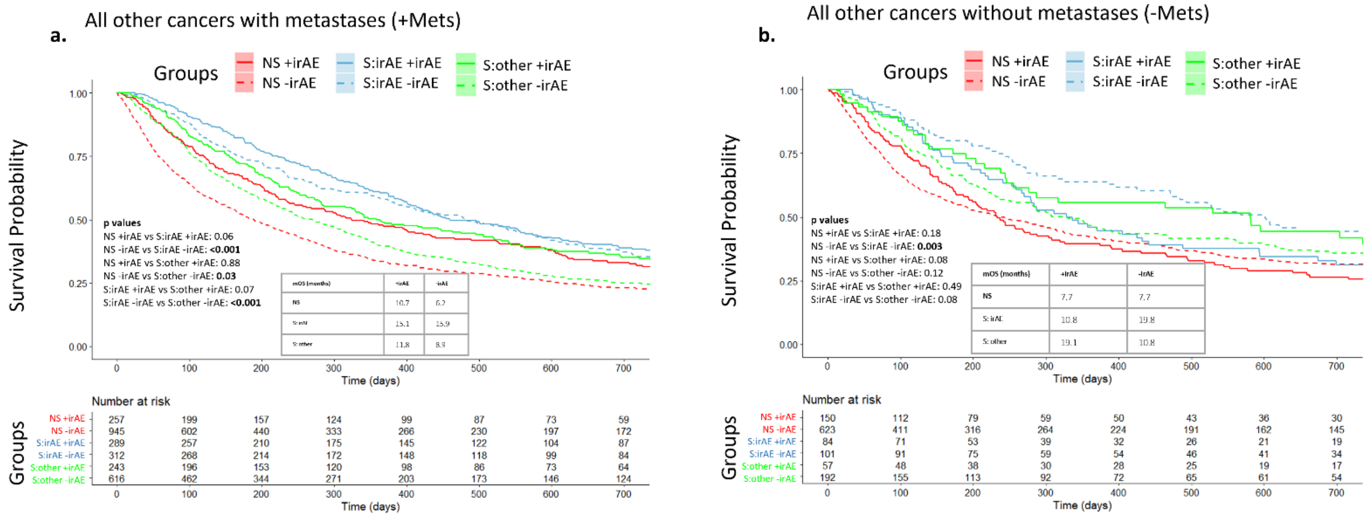

Kaplan-Meier curves showing association between steroid use ~~on~~ with overall survival in patients with all remaining cancers (outside of lung, melanoma, and urinary tract) with and without secondary metastases. Survival probability shown on y-axis over time (in days) on x-axis. Table below graphs demonstrate remaining number of patients at risk over 8 time points. No steroid (NS) group is represented in red while patients who received steroids for irAE (S:irAE) vs non-irAE (S:other) are represented in blue and green, respectively. +/- irAE ICD codes represented by solid and dashed lines, respectively. Median OS in months listed in associated tables. Adjusted significance threshold $p < 0.003$. **a.** Patients with secondary metastases. **b.** Patients without secondary metastases.

© 2023 Van Buren I et al. *JAMA Network Open.*

eFigure 10. Predominant steroid type and survival

a. Full steroid cohort

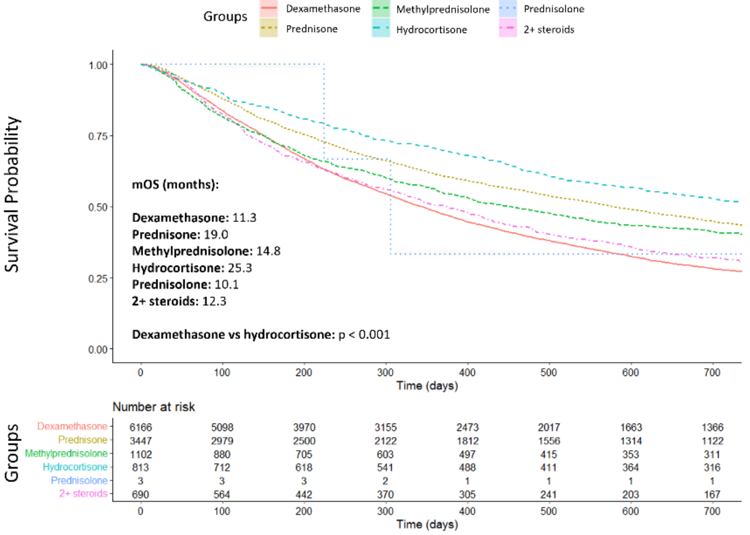

b. S:irAE

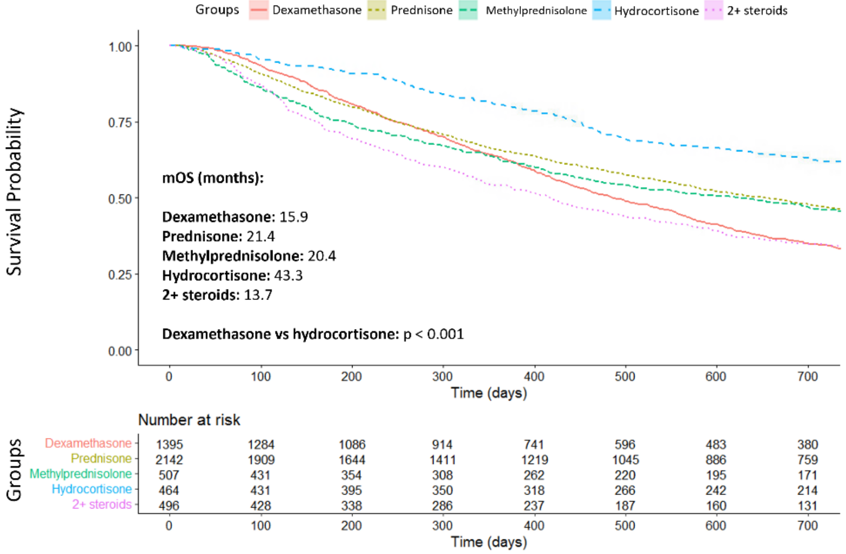

c. S:other

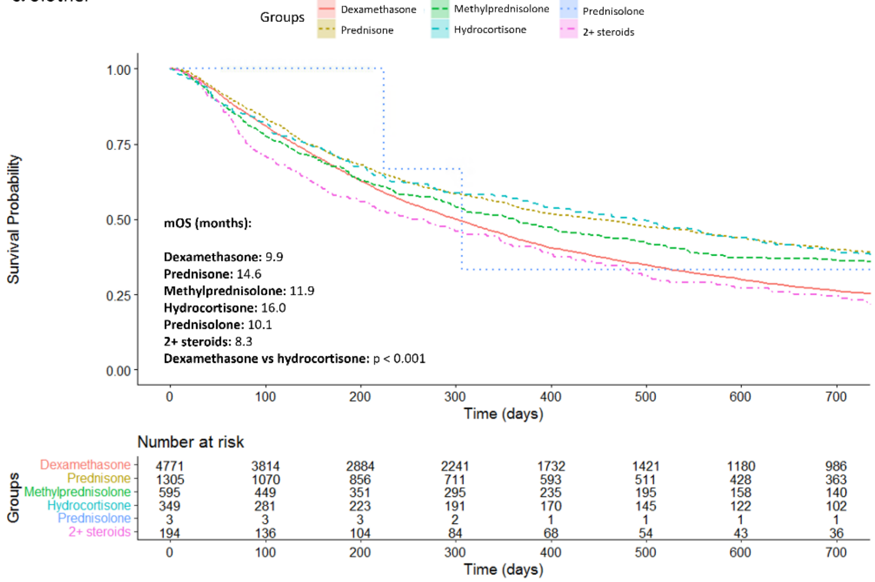

© 2023 Van Buren I et al. *JAMA Network Open.*

Kaplan-Meier curves showing association between the predominant steroid that patients received during ICI treatment with overall survival. 2+ steroids refers to patients who received multiple steroids in equal proportion. Survival probability shown on y-axis over time (in days) on x-axis. Table below graphs demonstrate remaining number of patients at risk over 8 time points. Median overall survival (mOS) in months listed. Adjusted significance threshold p < 0.003. **a.** Full steroid cohort. **b.** Patients with irAE associated steroid pattern of use (S:irAE). **c.** Patients with non-irAE associated steroid pattern of use (S:other).

eFigure 11. ICI target and steroid use on survival across cancer types

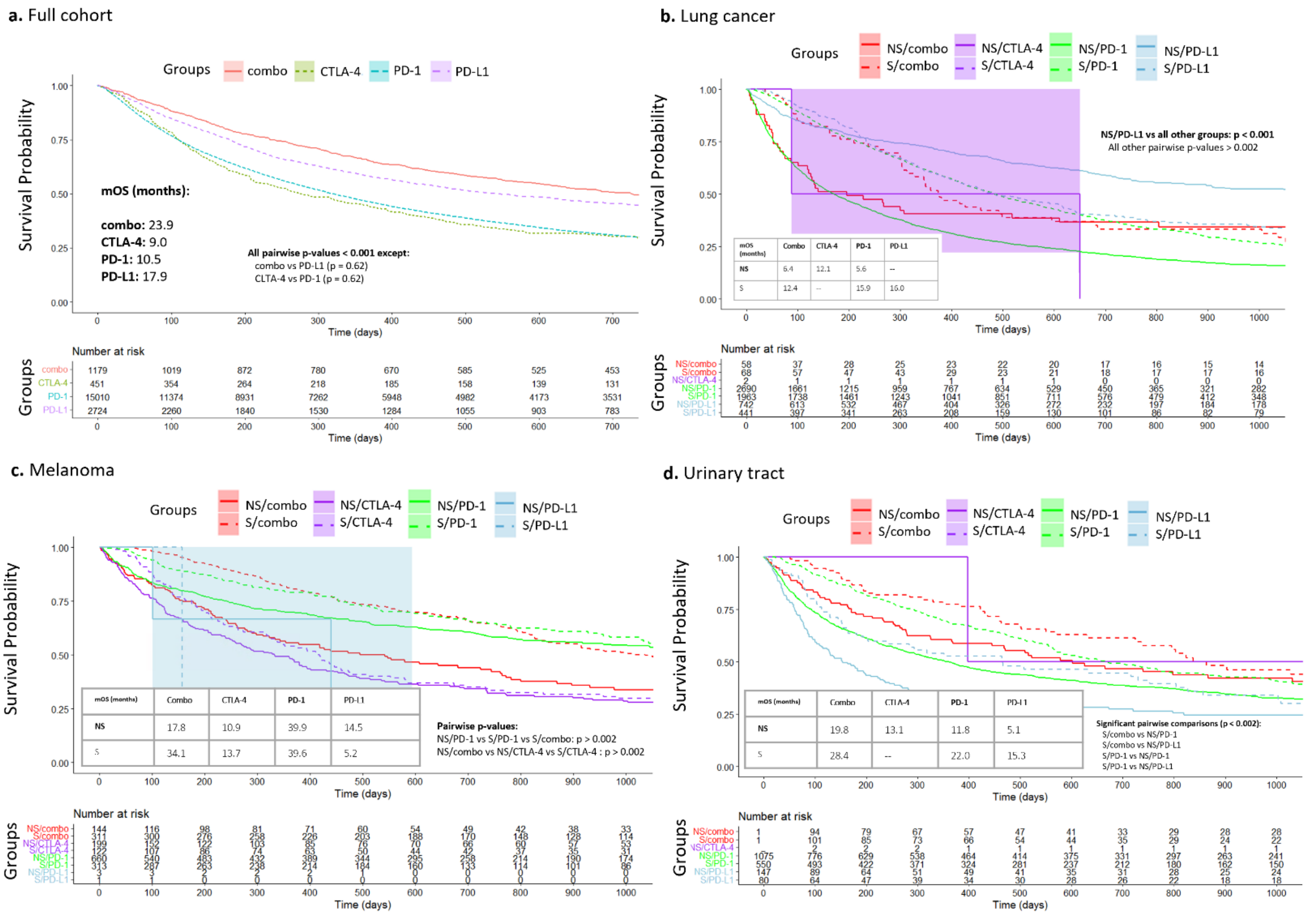

Kaplan-Meier curves showing association between ICI targets (combo: combination therapy with CTLA-4 and PD-(L)1 inhibitors, PD-1: anti-PD-1 monotherapy, PD-L1: anti-PD-L1 monotherapy) with overall survival, stratified by cancer type and presence of irAE associated steroid use. Survival probability shown on y-axis over time (in days) on x-axis. Table below graphs demonstrate remaining number of patients at risk over varying time points. Median overall survival (mOS) in months listed. In parts b-d, NS refers to no steroid patients (solid line) and S refers to patients with irAE associated steroid use (S:irAE subgroup, dashed line). **a.** ICI targets on survival outcomes in full cohort. Adjusted significance threshold p < 0.008. **b.** Survival outcomes in lung cancer patients, stratified by ICI target and steroid use. **c.** Survival outcomes in melanoma patients, stratified by ICI target and steroid use. **d.** Survival outcomes in urinary tract cancer patients, stratified by ICI target and steroid use. Adjusted significance threshold for parts b-d p < 0.002.

© 2023 Van Buren I et al. *JAMA Network Open.*

eFigure 12. Steroid type and survival in lung cancer patients receiving anti-PD-L1 monotherapy

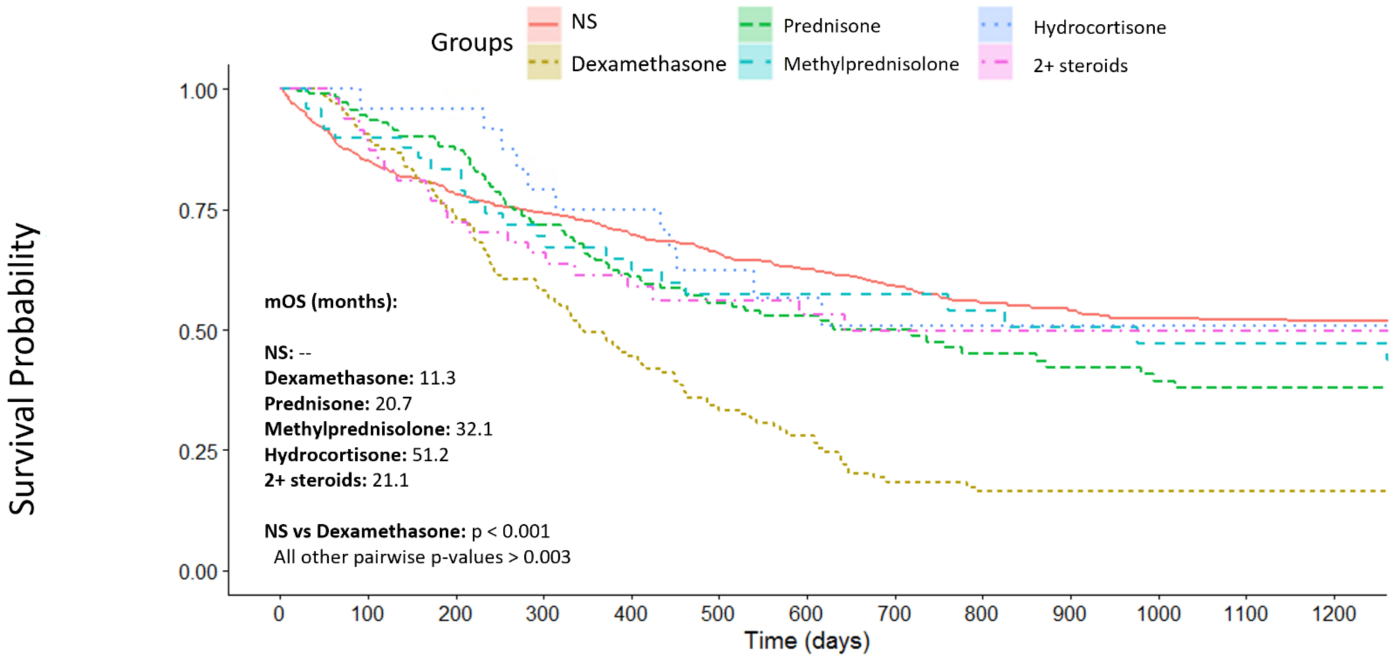

Number at risk

Groups	0	100	200	300	400	500	600	700	800	900	1000	1100	1200
NS	742	613	532	467	404	326	272	232	197	184	178	176	174
Dexamethasone	130	117	95	74	53	38	32	19	17	17	17	17	17
Prednisone	189	172	153	112	86	66	53	44	33	30	28	27	27
Methylprednisolone	49	43	36	29	27	23	19	18	16	15	14	14	14
Hydrocortisone	25	23	23	19	18	13	10	9	9	9	9	9	9
2+ steroids	48	42	34	29	24	19	16	11	11	11	11	11	11

Time (days)

Kapan-Meier curve showing association between steroid type and survival outcomes in lung cancer patients receiving anti-PD-L1 monotherapy. Survival probability shown on y-axis over time (in days) on x-axis. Table below graphs demonstrate remaining number of patients at risk over varying time points. Median overall survival (mOS) in months listed. NS refers to no steroid patients. Adjusted significance threshold p < 0.003.

© 2023 Van Buren I et al. *JAMA Network Open.*

eFigure 13. Steroid timing and ICI continuation status on survival in S:other patients

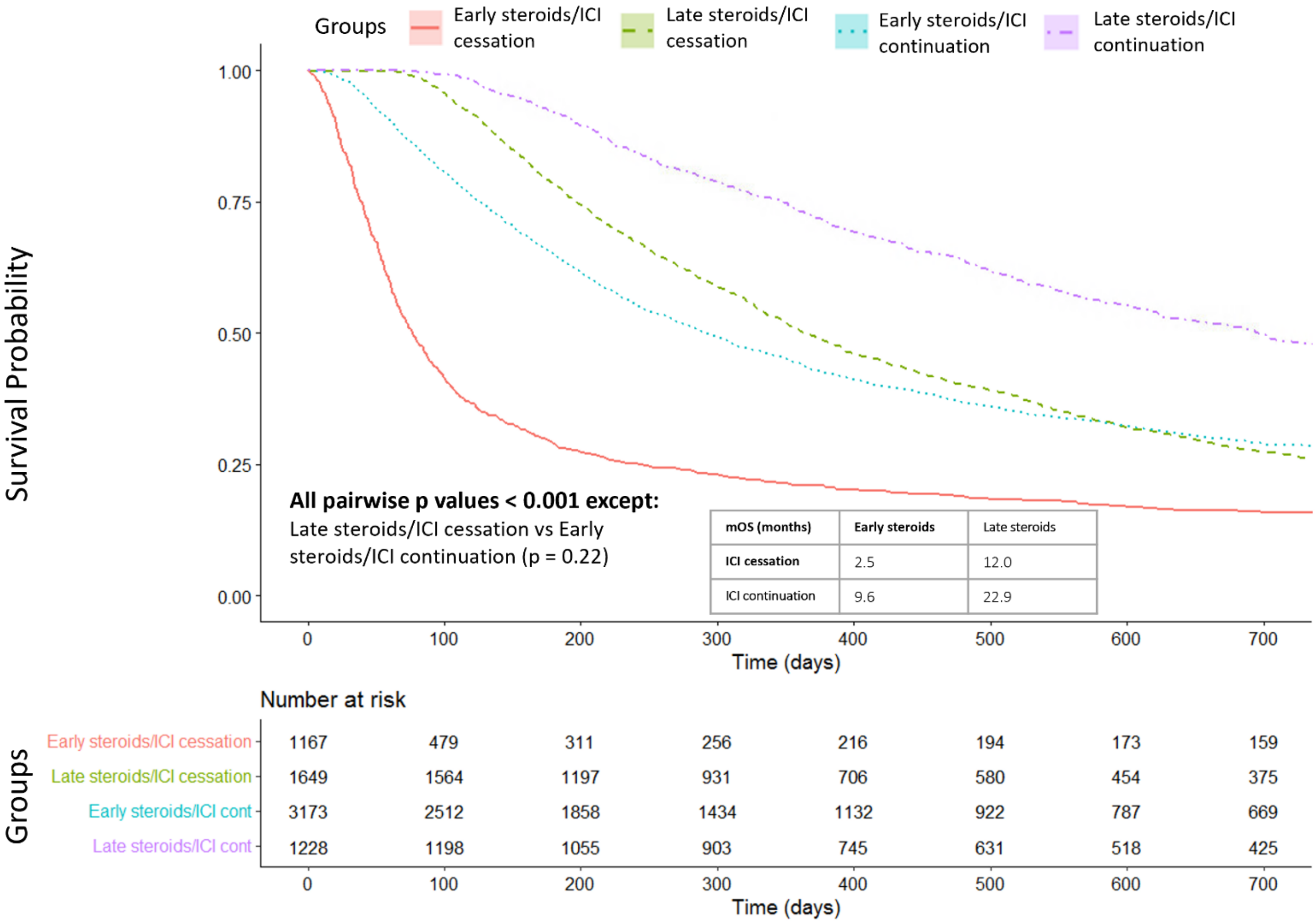

Kaplan-Meier curve demonstrating association between steroid timing and ICI continuation status with overall survival in patients with non-irAE associated steroid use (S:other, n = 7217). Early and late steroid use is defined as steroids administered <2 or ≥2 months after ICI initiation. ICI status defined as continuation or cessation of ICI treatment after steroid initiation. Survival probability shown on y-axis over time (in days) on x-axis. Table below graph demonstrate remaining number of patients at risk over 8 time points. Early steroids/ICI cessation group in red, late steroids/ICI cessation group in green, early steroid/ICI continuation group in blue, and late steroids/ICI continuation group in purple. Median OS in months listed in table. Adjusted significance threshold < 0.008.

© 2023 Van Buren I et al. *JAMA Network Open.*
