# Supplementary material for: Survival Among Veterans Receiving Steroids for Immune-Related Adverse Events After Immune Checkpoint Inhibitor Therapy
Source: JAMA Netw Open. 2023 Oct 31;6(10):e2340695. doi: 10.1001/jamanetworkopen.2023.40695 (PMC10618850; doi:10.1001/jamanetworkopen.2023.40695)
Supplement: Supplement 2. — Data Sharing Statement [file jamanetwopen-e2340695-s002.pdf]

Data Sharing Statement

Van Buren. Survival Among Veterans Receiving Steroids for Immune-Related Adverse Events After Immune Checkpoint Inhibitor Therapy. *JAMA Netw Open*. Published October 31, 2023. doi:10.1001/jamanetworkopen.2023.40695

Data
Data available: No

Additional Information
Explanation for why data not available: Data can be made available to qualified VINCI researchers.
